# PAD-Net: a lightweight end-to-end detector for pepper anthracnose in complex agricultural environments

**DOI:** 10.3389/fpls.2026.1878506

**Published:** 2026-08-21

**Authors:** Hualiang Lv, Heng Wang, Lin Song, Zicong Yan, Tianyu Li, Wenyi Yin

**Affiliations:** 1 School of Mathematics and Computer Science, Wuhan Polytechnic University, Wuhan, China; 2 School of Computer Science, Wuhan University, Wuhan, China

**Keywords:** attention mechanism, deep learning, Mamba, object detection, pepper anthracnose, transformer

## Abstract

Peppers are globally significant economic crops, but their yield is severely threatened by anthracnose. Automated detection remains challenging due to irregular lesion boundaries, drastic scale variations, complex backgrounds, and dense foliage occlusion. To address these issues, this study proposes PAD-Net, a lightweight end-to-end detector. Its LGMambaNet backbone synergizes DSwin-Mamba for deformable sliding window-based local modeling with FA-WTMamba for global frequency-domain analysis. A C3K2_PC module is introduced to capture multi-directional edge signals, enhancing sensitivity to blurred and multi-scale features. Furthermore, a DSSA module is designed to suppress background interference. Evaluated on the Pepper Anthracnose Dataset using standard COCO metrics, PAD-Net significantly outperforms the baseline DEIM model. Specifically, the PAD-Net nano version achieves an *AP* of 0.430, an *AP*_50_ of 0.846, and an *AP*_75_ of 0.382, representing a notable improvement over the baseline. The PAD-Net small version further attains an *AP* of 0.446, an *AP*_50_ of 0.861, and an *AP*_75_ of 0.404, delivering a substantial gain of 3.0% in *AP* compared to the baseline while also reducing model parameters. Generalization tests on Tomato Leaf, FieldPlant, and IP102 datasets further validate its superior detection capabilities and feature extraction. This research achieves an optimal balance between precision and efficiency, providing a reliable solution for automated monitoring and precision crop protection in smart agriculture.

## 1 Introduction

Peppers are among the most globally significant and economically vital crops, constituting a fundamental component of the international food supply chain (Zou and Zou, 2021). However, pepper production is severely threatened by anthracnose. This destructive disease, primarily caused by Colletotrichum species, spreads rapidly in warm and humid environments and leads to irreversible fruit rot (Ridzuan et al., 2018). In China, anthracnose causes yield losses of up to 50% in affected regions, resulting in hundreds of millions of dollars in economic damage annually (Li and Han, 2026). Therefore, early and effective detection is imperative for mitigating disease spread and safeguarding agricultural productivity.

Traditional manual inspection in agriculture faces significant challenges. Its reliability is often unstable due to the subjective judgment of inspectors and is particularly susceptible to harsh weather, such as rain or fog. This labor-intensive approach cannot support large-scale monitoring or real-time decision-making in modern agricultural systems. Moreover, rising labor costs and an aging rural population make manual detection unsustainable (Zhai et al., 2020; Zhou et al., 2026). Fortunately, the rapid development of machine learning and artificial intelligence has expanded the use of computer vision in smart agriculture (Akkem et al., 2023; Karunathilake et al., 2023). These technologies provide new and efficient pathways for precision agriculture.

In the domains of machine learning and early deep learning, systematic research has focused on identifying pepper diseases and pests with varying degrees of success. Thirumalraj et al. (Thirumalraj et al., 2024) proposed a hybrid framework integrating the TJO algorithm with MSTNet to achieve a classification accuracy of 99.2% on the PlantVillage dataset. To address sample imbalance, Su et al. (Su et al., 2019) developed a CNN-LSVM hierarchical model that optimizes performance through a cost-sensitive Local Support Vector Machine. Additionally, Chen et al. (Chen et al., 2023) enhanced feature extraction for insect pests on chili plants by integrating HSV-based color features into CNN frameworks, while Cetinkaya et al. (Çetinkaya and Tandirovic Gursel, 2025) introduced Pep-VGGNet, utilizing color enhancement and frozenlayer fine-tuning to improve diagnostic speed across multiple disease types. Despite these advancements, traditional methods often rely on constrained representational capacities, leading to poor adaptability in complex, dynamic field environments and limited generalization across diverse farm scenarios.

In the landscape of modern neural architectures, the YOLO series (Wang et al., 2024a; Khanam and Hussain, 2024; Tian et al., 2025; Lei et al., 2025; Sapkota et al., 2025) has emerged as a primary framework for agricultural detection due to its superior balance of speed, precision, and lightweight deployment. Mathew et al. (Mathew and Mahesh, 2022) successfully applied YOLOv5 to early bacterial spot detection in bell pepper leaves while reducing model parameters by 90%, significantly enhancing feasibility for mobile deployment. Furthermore, Yue et al. (Yue et al., 2024) introduced YOLOv7-GCA, which incorporates GhostNetV2 and CBAM modules to facilitate real-time field detection on Android devices. More recently, Wang et al. (Wang et al., 2025) proposed YOLO-Pepper based on YOLOv10n to address small-target and occlusion issues in greenhouse environments. However, inherent architectural constraints in the YOLO series remain; specifically, the reliance on Non-Maximum Suppression (NMS) for post-processing frequently leads to missed or false detections in dense or heavily occluded scenes, and robustness against backgrounds with high color similarity requires further improvement.

By way of comparison, the Real-Time DEtection TRansformer (RT-DETR) proposes a streamlined methodology that effectively eliminates the dependence on NMS through Hungarian matching and a global attention mechanism (Zhao et al., 2024). The hybrid encoder structure of RT-DETR efficiently processes multi-scale features, while its IoU-aware query selection enhances localization precision. Li et al. (Li et al., 2025) developed GP-DETR, which integrates a GELAN backbone and BiE-FPN to optimize detection under similar-colored backgrounds and occlusions. Additionally, Wang et al. (Wang et al., 2024b) developed the PD-TR model based on an enhanced DINO Transformer, demonstrating superior performance across expansive data repositories covering six crops, including peppers, and outperforming traditional CNN baselines in perceiving diverse crop diseases.

Despite the aforementioned advancements, achieving high-precision and robust detection of pepper anthracnose in real-world agricultural environments still encounters several formidable challenges. Specifically, the lesions exhibit a dramatic scale span, ranging from minute early-stage spots to large-area fused necrotic regions. These characteristics, coupled with complex color transitions and highly irregular, diffuse boundary features, significantly increase the difficulty of feature extraction and precise boundary delineation. Furthermore, the detection process is often hampered by complex field backgrounds and high-similarity interference. These difficulties are compounded by significant foliage occlusion and fruit overlap in high-density cultivation, which leads to fragmented visual cues and challenges the model’s ability to localize partially obscured targets.

Seeking to overcome these challenges, the current investigation introduces Pepper Anthracnose Disease Net (PAD-Net), a high-performance and lightweight end-to-end detector. The core of our architecture is the self-designed LGMambaNet backbone, which leverages a hybrid global-local modeling mechanism to effectively handle occluded lesions and irregular boundaries. Furthermore, we introduce the C3K2_PC neck module to capture multi-directional edge signals, enhancing the model’s sensitivity to blurred lesion gradients. To suppress redundant background interference, the DSSA (Dual-Statistics Synergistic Attention) module is deployed at the output stage, performing recalibration of spatial and channel features.

The main contributions of this paper are summarized as follows:

- Construction of the Pepper Anthracnose Dataset for complex field environments. A robust dataset was established by curating high-quality images and employing the Style-aware Mamba (SAMAM) model to simulate adverse weather conditions. This approach provides a dependable data base for verification of architectural robustness under various agricultural scenarios.
- Design of LGMambaNet, a lightweight backbone integrating DSwin-Mamba for local modeling and FA-WTMamba for global frequency analysis. This architecture simultaneously captures the edge details of irregular lesions and facilitates the establishment of long-distance spatial modeling while ensuring computational efficiency for resource-constrained agricultural environments.
- Proposal of PAD-Net, a high-precision model combining LGMambaNet, the C3K2_PC neck, and the DSSA attention module. This integration improves sensitivity to lesion boundaries and suppresses background noise, delivering superior performance on the Pepper Anthracnose Dataset while demonstrating highly competitive generalization performance, particularly in terms of overall average precision, on external benchmarks.

## 2 Materials and methods

### 2.1 Dataset materials and preparation

To ensure the scientific rigor of the evaluation, four distinct agricultural datasets are utilized in this study. The primary focus is the Pepper Anthracnose Dataset, which is employed for the fundamental training and performance validation of the proposed PAD-Net. To facilitate a more exhaustive appraisal of the robustness inherent in the proposed framework, the Tomato Leaf and FieldPlant public datasets are used to evaluate the cross-crop generalization performance and robustness of the PAD-Net detector under severe real-world environmental disturbances (Imtiaz et al., 2025; Moupojou et al., 2023). Additionally, the IP102 dataset serves as a benchmark to assess the feature extraction efficiency and representation capability of the LGMambaNet backbone across diverse agricultural vision tasks (Wu et al., 2019).

#### 2.1.1 Pepper Anthracnose Dataset

The Pepper Anthracnose Dataset was established by curating high-quality images from public platforms, specifically the Roboflow (yolov7project, 2023) and Kaggle (Ansari, 2022) repositories. A total of 795 raw images were selected to form the foundational data, focusing on a single object class: anthracnose. Prior to annotation, duplicate and near-duplicate images were eliminated via MD5 checksums and manual screening to ensure sample independence. Bounding boxes were labeled using LabelImg by two graduate researchers under the guidance of senior researchers. For quality control, senior experts carefully reviewed and verified a portion of the annotated samples, systematically correcting or discarding mislabeled or ambiguous boxes. The dataset features lesions with irregular boundaries and complex color variations on pepper fruits.

**Figure 1:**
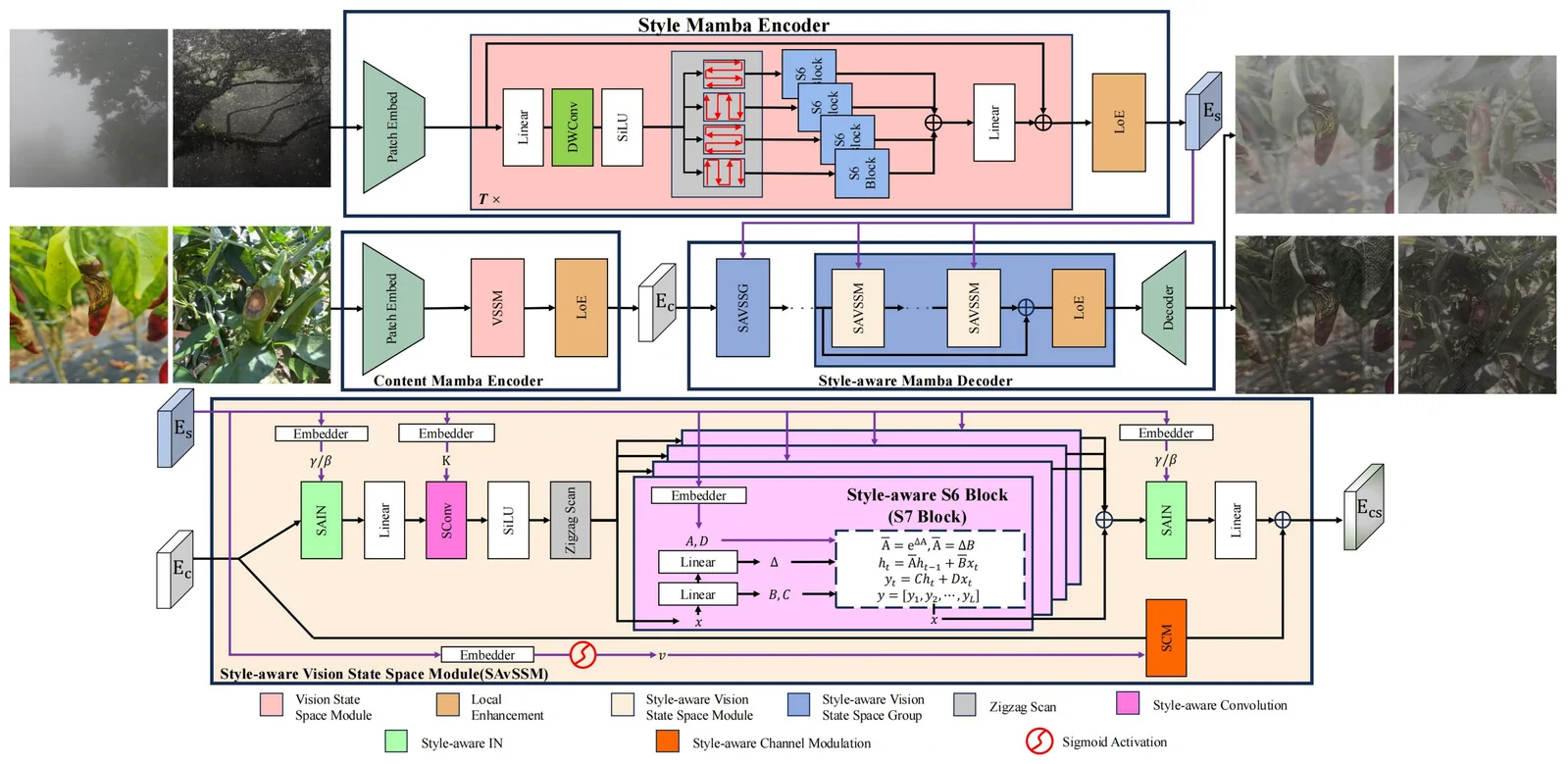
Architecture of the SAMAM model used for weather simulation and feature transfer.

**Figure 2:**
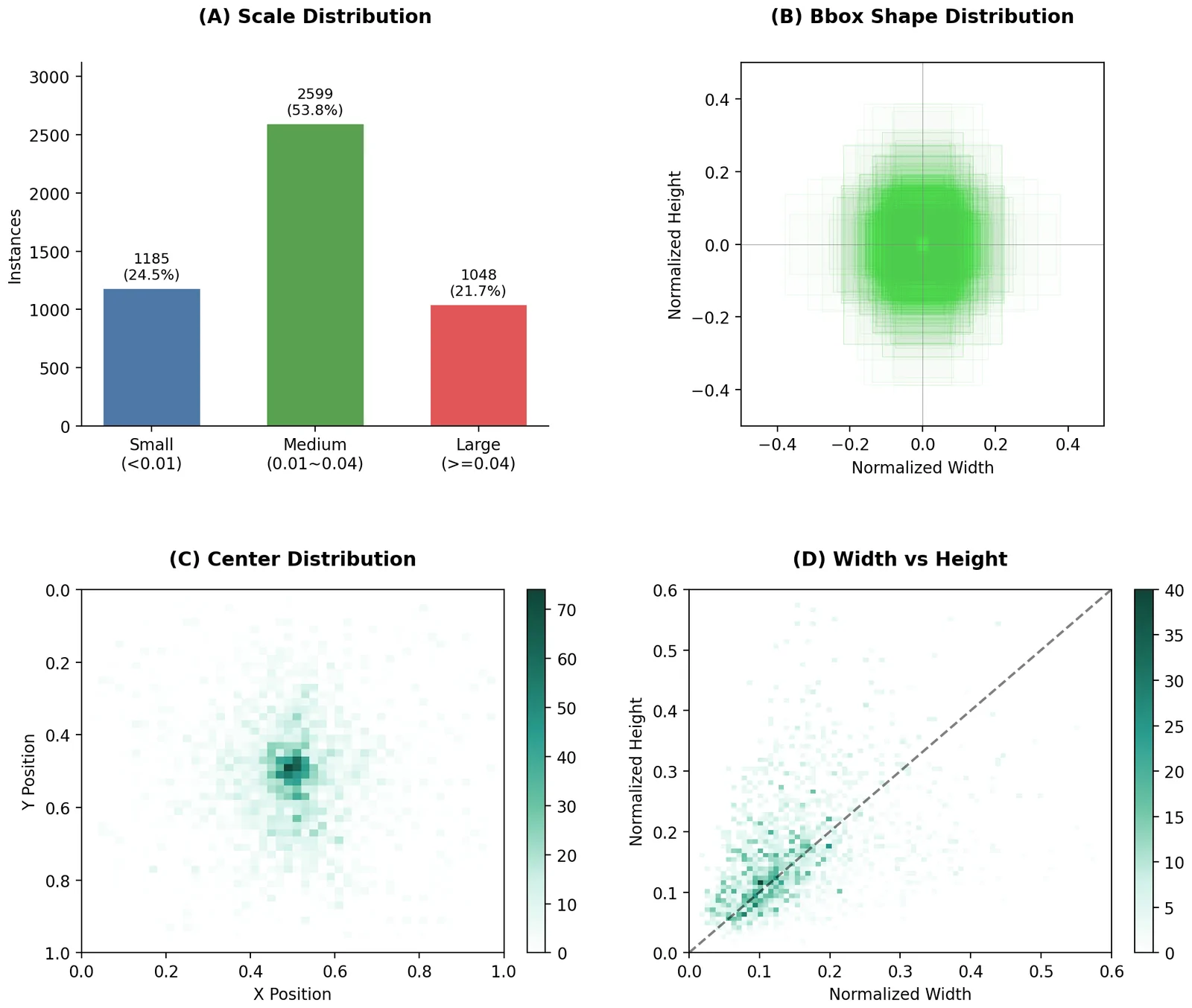
Statistical analysis diagram of the Pepper Anthracnose Dataset system: **(A)** scale distribution of bounding-box instances; **(B)** bounding-box shape distribution; **(C)** center distribution; and **(D)** width-versus-height distribution.

To provide a balanced assessment of detection accuracy, the curated dataset was first allocated into training and evaluation subsets at an 8:2 ratio. Subsequently, to enhance the model’s robustness against diverse field conditions and mitigate the risk of overfitting, a comprehensive data augmentation and preprocessing pipeline was executed exclusively on the training set. This included standard geometric preprocessing via Roboflow, such as image rotation and flipping, to increase spatial diversity. Furthermore, a feature transfer model, Style-aware Mamba (SAMAM) (Liu et al., 2025), was employed to simulate adverse weather environments. As shown in **Figure 1**, SAMAM effectively synthesizes dense fog and rainfall conditions, enabling the model to learn weather-invariant pathological features. By integrating these geometric and environmental augmentation strategies, the training dataset scale underwent a fivefold expansion, where each original image was transformed into five unique augmented samples. This process significantly enriched the diversity of the training data and mitigated the risk of overfitting to specific environmental lighting or weather conditions. Detailed statistical analysis regarding the lesion distribution and bounding box characteristics of the augmented dataset is illustrated in **Figure 2**.

#### 2.1.2 Generalization and benchmark datasets

To verify the generalization capability and backbone efficiency of the proposed architecture, the following three benchmark datasets were introduced.

The Tomato Leaf Dataset contains 731 high-resolution images and 1,621 annotations across seven categories: early blight, black spot, late blight, leaf mold, bacterial spot, target spot, and healthy leaves. It serves as a rigorous benchmark for multi-class disease detection under complex natural backgrounds.

FieldPlant is a field-conditioned plant disease benchmark comprising 5,170 field-captured images and 8,629 bounding box annotations across 27 disease classes of corn, cassava, and tomato. Serving as a public benchmark to evaluate model robustness in complex agricultural scenarios, its complex and adverse natural field environments pose severe challenges for object detection and feature discrimination.

IP102 is a large-scale insect pest benchmark comprising over 75,000 classification images and approximately 19,000 annotated detection images across 102 categories, organized hierarchically over field and economic crops. Its long-tail distribution and high intra-class variance pose significant challenges for backbone feature discrimination and representation.

### 2.2 Overall architecture of PAD-Net

**Figure 3:**
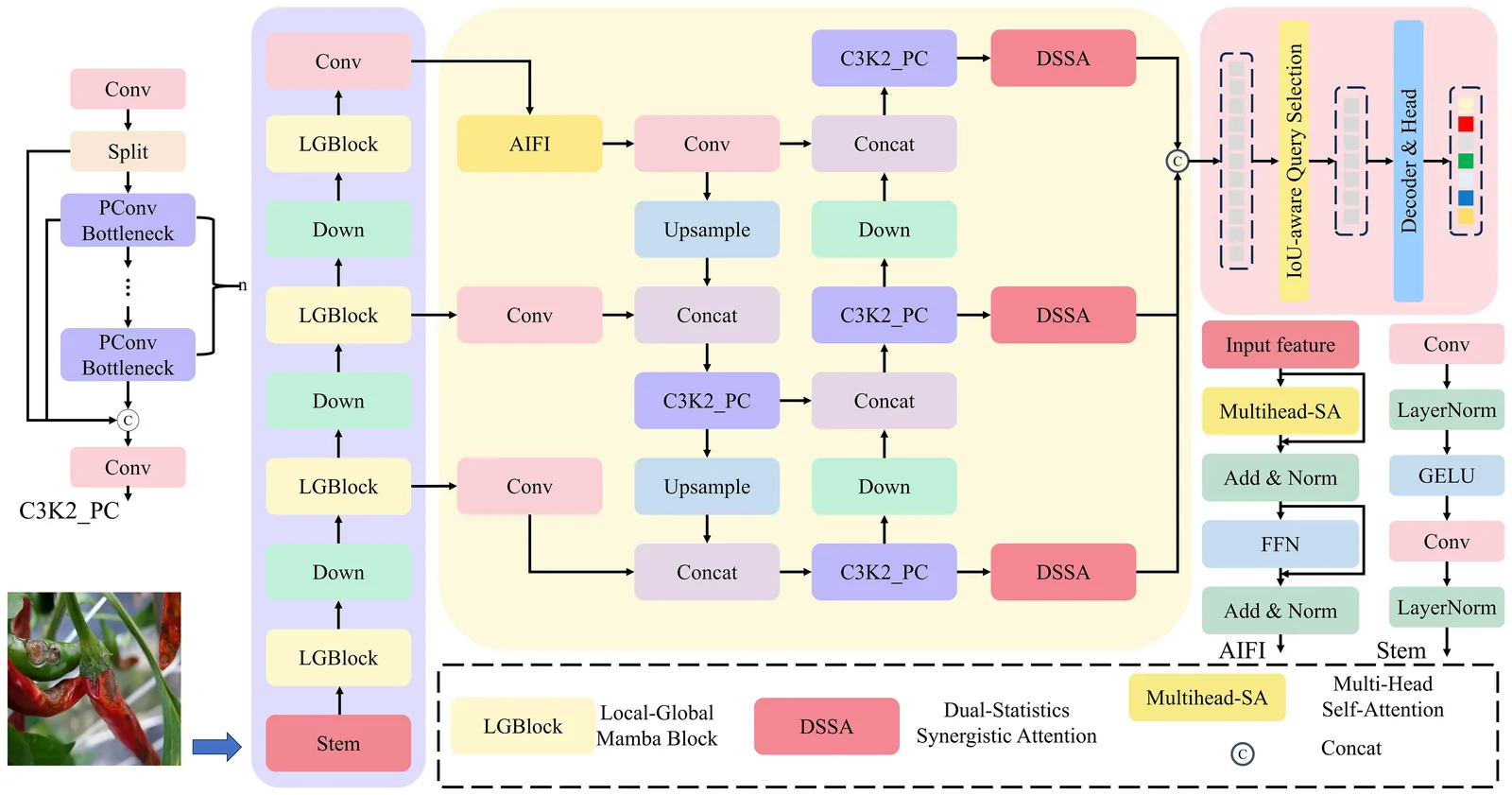
PAD-Net model overall architecture diagram.

In this study, PAD-Net is introduced, and its overall architecture is shown in **Figure 3**, aiming to overcome complex backgrounds and great changes of target scale in pepper anthracnose detection task. Drawing from the design of the DETR with Improved Matching (DEIM) (Huang et al., 2025b), our study develops PAD-Net as an efficient one-stage detection model. Choosing DEIM as the baseline framework, it has effective equilibrium between detection accuracy and the computational parameters. The architecture uses a modular design, comprising an efficient backbone, a bidirectional fusion neck, and a deeply optimized detection head. One of the main advantages of DEIM is that it uses a dense one-to-one matching strategy, which improves the standard Hungarian matching process. This strategy enables the allocation of the most appropriate prediction queries to each target, thereby effectively capturing small targets in complex backgrounds. Furthermore, the architecture can automatically change the importance of poor quality samples by introducing a loss function sensitive to matching quality. This method significantly improves the reliability of the model in the real farm environment.

In the specific implementation of PAD-Net, the backbone utilizes the self-designed LGMambaNet (shown on the left of **Figure 3**). By synergizing a hierarchical Stem module with multiple LGBlocks, detailed lesion features are extracted from pepper images. Subsequently, the multi-scale feature flow enters the feature fusion neck. At this stage, the model first employs the AIFI module to facilitate full-scale feature interaction, thereby enhancing spatial context modeling capabilities. To further improve feature fusion efficiency, the C3K2_PC module is introduced in the neck section. Its core mechanism employs Pinwheel-shaped Convolution (PConv) (Yang et al., 2025), which features a four-way parallel asymmetric sampling structure. PConv significantly expands the effective receptive field while substantially reducing parameter overhead. Moreover, its receptive field intensity distribution is highly compatible with the spatial pixel characteristics of pepper anthracnose lesions. This targeted design strengthens the model’s ability to capture multi-scale boundary features, effectively improving the recognition accuracy of tiny lesions amidst complex agricultural backgrounds during the feature fusion stage.

To suppress background interference arising from pepper leaf and fruit peel textures, the DSSA module is deployed at the output stage following the neck. This module guides the model to focus on the lesion regions by performing sequential recalibration of spatial and channel features. Finally, the fused and enhanced features are fed into the detection head, where high-quality initial query tokens are filtered through an IoU-aware Query Selection strategy. The Decoder and Head modules then work in coordination to output accurate category predictions and bounding box coordinates.

### 2.3 LGMambaNet backbone

As shown in **Figure 3**, the LGMambaNet backbone uses a standard four-stage hierarchical design, constructed by alternating LGBlocks and downsampling modules. Starting with an input image $I\in {\mathbb{R}}^{H\times W\times 3}$, the first stage begins with a Stem module—consisting of two successive 3 × 3 convolutions (stride=2) interleaved with Layer Normalization (LN) and GELU activation functions—which converts the image into a *H/*4×*W/*4 feature map having *C*_1_ channels. Subsequently, the backbone operates across four stages at scales of {*H/*4*,H/*8*,H/*16*,H/*32}, with corresponding channel dimensions of {*C*_1_*,C*_2_*,C*_3_*,C*_4_} and block configurations of {*D*_1_*,D*_2_*,D*_3_*,D*_4_}. For the subsequent three stages, downsampling is performed by a module composed of an LN followed by a 3 × 3 convolution with a stride of 2.

**Figure 4:**
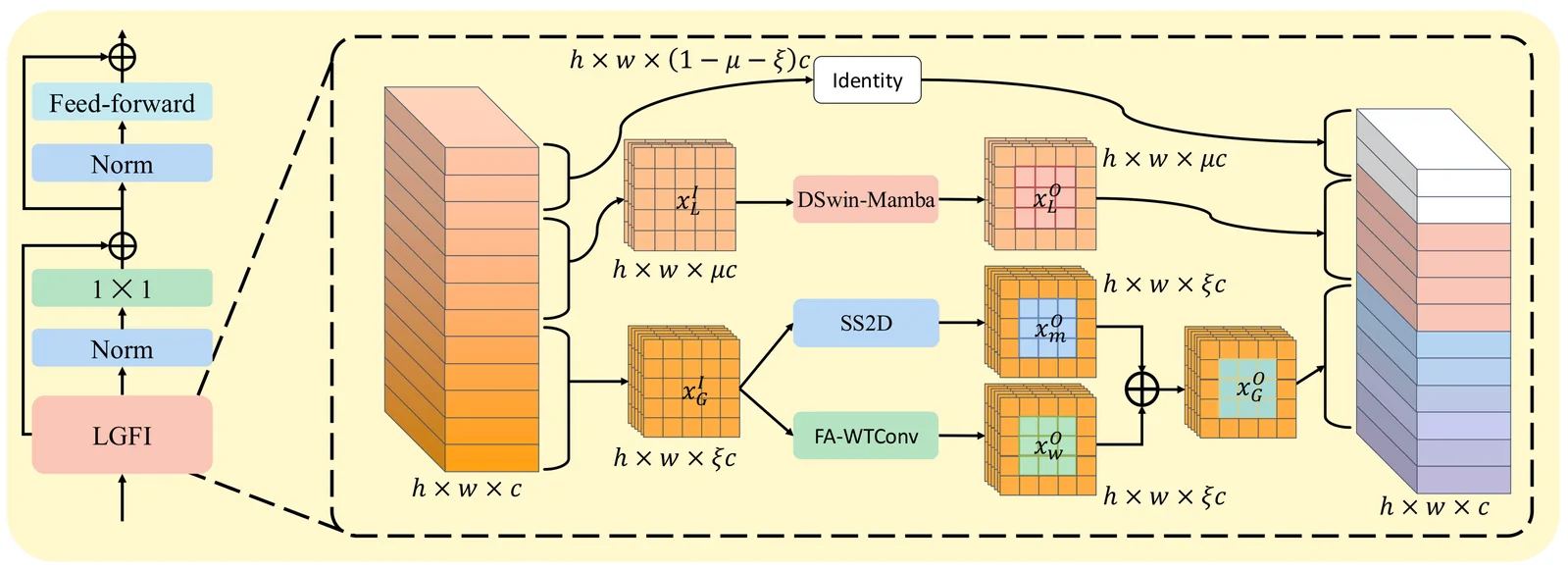
The structure of LGBlock.

As the primary computational unit, the architectural core of the LGBlock comprises a Local-Global Feature Interaction (LGFI) module and a Feed-Forward Network (FFN), as shown in **Figure 4**. For an input feature $X\in {\mathbb{R}}^{BesC\times H\times W}$, the residual processing flow is defined as Equations 1, 2:

(1) $$X^{\prime }=X+\text{LGFI}\left(\text{LN}\left(X\right)\right)$$

(2) $$X^{\prime \prime }=X^{\prime }+\text{DropPath}\left(\gamma \cdot \text{FFN}\left(\text{LN}\left(X^{\prime }\right)\right)\right)$$

The LGFI module is responsible for achieving cross-scale feature alignment and fusion. Observing the right section of **Figure 4**, the architectural design adopts a channel-partitioning strategy optimized for efficient feature interaction (He et al., 2025), where the input channels are partitioned into distinct subsets and processed independently by three parallel paths: an identity mapping path that preserves the original features, a Deformable Sliding Window Mamba (DSwin-Mamba) path focused on the finegrained modeling of local details, and a Frequency-Aware Wavelet Transform Mamba (FA-WTMamba) path. This latter path integrates the 2D Selective Scan (SS2D) (Liu et al., 2024) featuring bidirectional scanning mechanisms and FA-WTConv, which is responsible for the efficient modeling of global context features. This parallel multi-branch design ensures both numerical stability and discriminative power when processing the complex lesion features of pepper anthracnose.

Under this architecture, each branch is processed independently by its exclusive operator: FA-WTMamba is utilized for global frequency-domain modeling, DSwin-Mamba captures local geometric features, and the *C*_identity_ path employs identity mapping to bypass redundant computation for agricultural background textures, such as smooth fruit peels. Finally, the features from all branches are fused through channel concatenation and a convolutional projection, as formulated in Equations 3, 4:

(3) $$F_{\text{cat}}=\text{Concat}\left[\text{FA}-\text{WTConv}\left(X_{g}\right),\;\text{DSwin}-\text{Mamba}\left(X_{l}\right),\;X_{\text{id}}\right]$$

(4) $$Y=\text{LN}\left({\text{Conv}}_{1\times 1}\left(\text{ReLU}\left(F_{\text{cat}}\right)\right)\right)$$

The architectural specifications of the proposed LGMambaNet backbone are detailed in **Table 1**. The backbone employs a four-stage hierarchical design with a non-uniform repeat configuration of {2,3,7,2}, where Stage 3 (*D*_3_ = 7) is designated as the primary semantic bottleneck to enhance non-linear representation capacity. To optimize the trade-off between detection accuracy and computational efficiency, the global ratio *ξ_i_* is dynamically scheduled across stages. Specifically, *ξ_i_* is maintained at 0.8 in the shallow stages (Stages 1 and 2) to preserve fine-grained lesion boundaries, and is progressively reduced to 0.7 and 0.6 in the deeper stages (Stages 3 and 4, respectively) to prioritize high-level semantic abstraction. Furthermore, the channel dimensions scale progressively from 48 to 128. This expansion effectively compensates for the loss of spatial resolution during downsampling, ensuring that high information density is maintained throughout the hierarchical feature extraction process.

**Table 1:** Architecture configuration of the LGMambaNet backbone.

Layer	Output size	Output stride	Repeat	Channels	Global ratio *ξ*	Local ratio *μ*	Identity ratio
Image	H×W	1	—	3	—	—	—
Stem	$\frac{H}{4}\times \frac{W}{4}$	4	1	48	—	—	—
LGBlock	$\frac{H}{4}\times \frac{W}{4}$	4	2	48	0.8	0.2	0
Downsample	$\frac{H}{8}\times \frac{W}{8}$	8	1	64	—	—	—
LGBlock	$\frac{H}{8}\times \frac{W}{8}$	8	3	64	0.8	0.2	0
Downsample	$\frac{H}{16}\times \frac{W}{16}$	16	1	96	—	—	—
LGBlock	$\frac{H}{16}\times \frac{W}{16}$	16	7	96	0.7	0.2	0.1
Downsample	$\frac{H}{32}\times \frac{W}{32}$	32	1	128	—	—	—
LGBlock	$\frac{H}{32}\times \frac{W}{32}$	32	2	128	0.6	0.3	0.1

#### 2.3.1 FA-WTMamba global branch implementation

The FA-WTMamba module implements global spatial modeling and frequency-domain feature extraction through a dual-path parallel architecture. Given the input features of the global branch $X_{g}\in {\mathbb{R}}^{B\times C_{g}\times H\times W}$, the processing flow consists of an SS2D path and a Haar Wavelet Transform path. In the SS2D path, a global context representation is constructed via bidirectional selective scanning, followed by a Squeezeand-Excitation (SE) module for channel feature recalibration, as formulated in Equation 5:

(5) $$X_{\text{ssm}}=\text{SE}\left(\text{SS}2\text{D}\left(X_{g}\right)\right)$$

Simultaneously, the wavelet path utilizes the Haar Discrete Wavelet Transform (DWT) to decompose *X_g_*into four frequency sub-bands: Low-Low (LL), Low-High (LH), High-Low (HL), and High-High (HH), following Equation 6:

(6) $$X_{\text{wt}}=\text{DWT}\left(X_{g}\right)\in {\mathbb{R}}^{B\times 4C_{g}\times \frac{H}{2}\times \frac{W}{2}}$$

Subsequently, Depthwise Convolution (DWConv) and a Squeeze-and-Excitation (SE) module are applied to the stacked sub-band features, which are then reconstructed to the original spatial resolution through the Inverse Wavelet Transform (IWT). This process is expressed as Equations 7, 8:

(7) $$X_{\text{wt}}{\mathrm{''}}^{=}\text{SE}\left(\text{DWConv}\left(X_{\text{wt}}\right)\right)$$

(8) $$X_{\text{rec}}=\text{IWT}\left(X_{\text{wt}}^{\prime }\right)$$

Finally, the output of the global branch is fused by adding the features from both paths via a residual connection, as shown in Equation 9:

(9) $$X_{g}^{\prime }=X_{\text{ssm}}+X_{\text{rec}}$$

**Figure 5:**
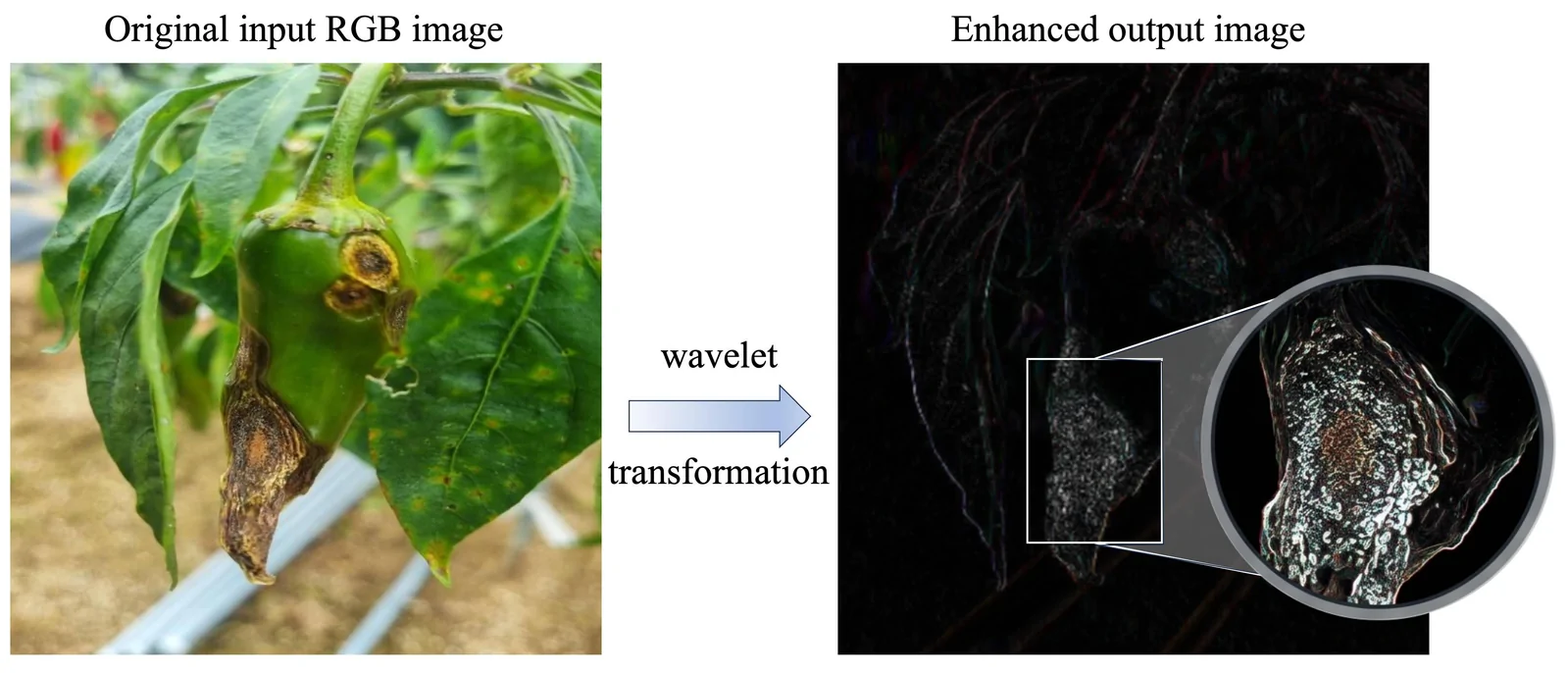
Haar wavelet transform visualization of a pepper anthracnose sample.

To intuitively verify the effectiveness of the wavelet transform in feature extraction for pepper anthracnose, **Figure 5** illustrates the visualization results of the HL sub-band of a typical sample after single-level Haar wavelet decomposition. In the original image, the boundary between the lesion and healthy tissue is relatively blurred due to color gradients. However, the HL sub-band clearly delineates the vertical gradient information of the lesion edges with high-intensity responses against a black background. This indicates that the wavelet transform can explicitly map lesion details, which are difficult to distinguish in the spatial domain, into frequency sub-bands, thereby effectively reducing interference from complex agricultural backgrounds and providing the model with more discriminative edge feature representations.

Based on these frequency-domain characteristics, FA-WTMamba adopts a dual-path architecture that jointly models global spatial dependencies and fine-grained spectral features. Specifically, the SS2D branch captures long-range spatial context with linear computational complexity, establishing global contextual consistency across the image. Concurrently, the wavelet branch decomposes spatial representations into distinct frequency sub-bands, explicitly preserving high-frequency boundary gradients that tend to be smoothed out in pure spatial feature extraction. By residually integrating global spatial context with spectral boundary cues, this dual-path design suppresses complex background clutter and enhances feature discriminability for low-contrast, diffuse lesions without incurring the heavy computational overhead of dense self-attention mechanisms.

#### 2.3.2 DSwin-Mamba local branch implementation

**Figure 6:**
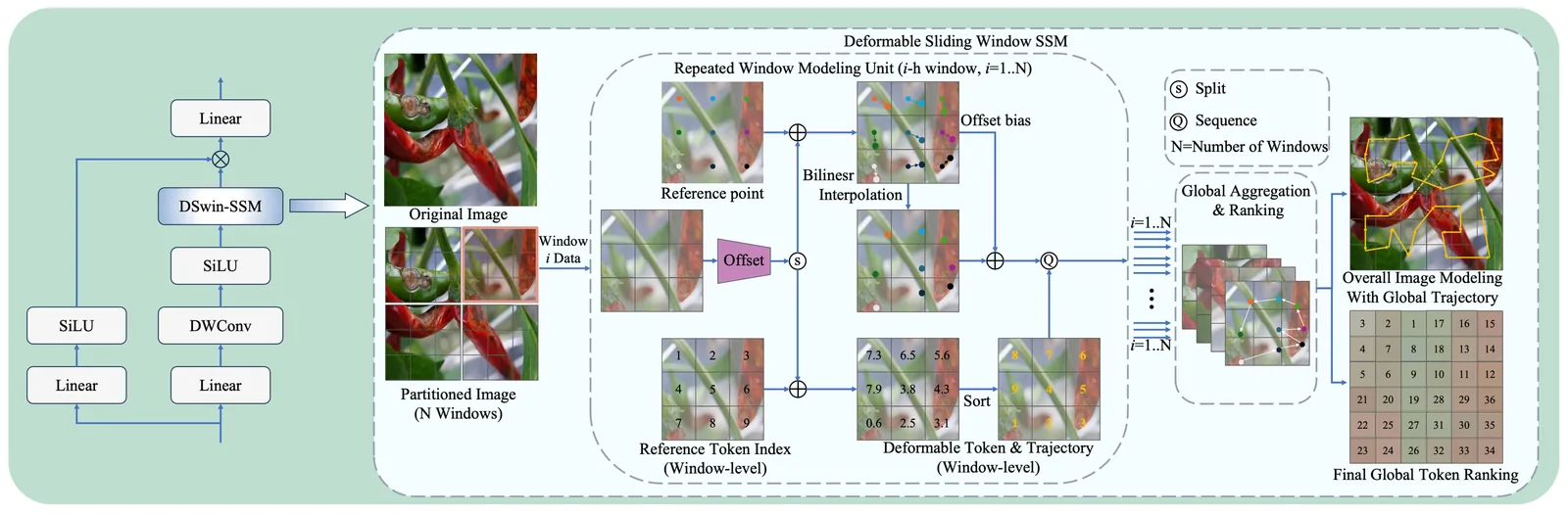
Module architecture and scanning strategy of DSwin-Mamba.

The local branch adopts the DSwin-Mamba block, which utilizes a Deformable Sliding Window SSM (DSwin-SSM) to perform content-adaptive scanning over fine-grained local structures. The overall architecture is illustrated on the left side of **Figure 6**.

Given the local feature map $X_{l}\in {\mathbb{R}}^{B\times C_{l}\times H\times W}$, the DSwin-Mamba block follows a gated SSM design. The input is first linearly projected and split into a content branch and a gating branch, as defined in Equation 10:

(10) $$\left[X_{ct},Z\right]=W_{in}X_{l},\;Z=\text{SiLU}\left(Z\right)$$

where $d_{e}=\left[\alpha C_{l}\right]$ is the expanded channel dimension controlled by SSM ratio *α*, with $W_{in}\in {\mathbb{R}}^{2d_{e}\times C}$ projecting the input into two branches of dimension *d_e_*each.

Specifically, the content branch feature *X_ct_* passes through a depthwise convolution before being processed by the DSwin-SSM module, yielding the output feature *X_defssm_*. Afterward, the gating mechanism fuses *X_defssm_* with the gating branch *Z*, and linearly projects the result back, as expressed in Equation 11:

(11) $$X_{l}^{\prime }=W_{out}\left(X_{defssm}⊙Z\right)$$

As shown in **Figure 6**, the spatial domain is first partitioned into *N* non-overlapping windows of size *i*×*i*. For each window, an OffsetNet predicts a 2-D spatial offset Δ*p* and a scalar token index offset Δ*t* for every reference token. Specifically, given window feature *X_i_*, the OffsetNet applies a depthwise convolution followed by a channel attention module, which is formulated as Equation 12:

(12) $$X_{ca}=\sigma \left(W_{2}\text{GELU}\left(W_{1}\text{GAP}\left(X_{i}\right)\right)\right),\;F_{off}=\text{GELU}\left(\text{LN}\left(\text{DWConv}\left(X_{i}\right)⊙X_{ca}\right)\right)$$

where $W_{1}\in {\mathbb{R}}^{C_{l}/r\times C_{l}}$ and $W_{2}\in {\mathbb{R}}^{C_{l}\times C_{l}/r}$ are the linear projection weights with a reduction ratio *r* = 16.

Finally, a 1 × 1 convolution projects the offset feature *F_off_* into a 3-channel offset map, which is split into the 2-D spatial offset Δ*p* and the scalar token index offset Δ*t*, following Equation 13:

(13) $$\left[\text{Δ}p,\text{Δ}t\right]=\text{Split}\left(\text{tanh}\left({\text{Conv}}_{1\times 1}\left(F_{off}\right)\right)\right)$$

where tanh constrains both offsets to (−1,1). The channel attention suppresses redundant background noise and produces offset signals aligned with lesion boundaries.

Given Δ*p*, each reference token at *p*_0_ is relocated to $\hat{p}=p_{0}+\text{Δ}p$. Features are extracted via bilinear interpolation, with a learnable positional bias compensating for spatial displacement, as shown in Equation 14:

(14) $${\hat{X}}_{l}=\phi \left(X_{l},\hat{p}\right)+\phi \left(R,\hat{p}\right)$$

The deformable tokens from all *N* windows are then sorted by Δ*t* to form a content-adaptive 1-D sequence for SSM processing, with the inverse permutation recorded for spatial layout restoration. This mechanism dynamically steers the scanning trajectory toward lesion-boundary-dense regions, overcoming the limitations of fixed scanning schemes in finely capturing the blurred and outwardly diffusing transition zones of pepper anthracnose boundaries.

This adaptive local modeling is particularly critical in complex agricultural scenes, where anthracnose lesions rarely align with rigid window boundaries and are often obscured by foliage or adjacent fruits. By predicting spatial offsets and token order permutations from local feature content, DSwin-Mamba dynamically redirects its sampling grid toward structurally informative regions instead of following fixed, content-agnostic traversals. Consequently, this local branch complements the global frequencyaware module: while FA-WTMamba supplies broader contextual consistency and spectral boundary cues, DSwin-Mamba preserves fine-grained, spatially displaced local lesion details.

### 2.4 C3K2_PC neck

To further improve feature fusion efficiency and reduce computational overhead within the neck, the C3K2_PC module is proposed. Observing the left section of **Figure 3**, the module adopts a two-level nested Cross Stage Partial (CSP) structure. The input feature first undergoes a 1 × 1 convolution for channel projection, followed by a split operation to create two gradient paths. One path serves as a residual connection, while the other passes through a sequence of bottleneck blocks based on the PConv operator. Finally, the features from both paths are concatenated and fused via a transition layer to achieve comprehensive multi-scale spatial modeling.

**Figure 7:**
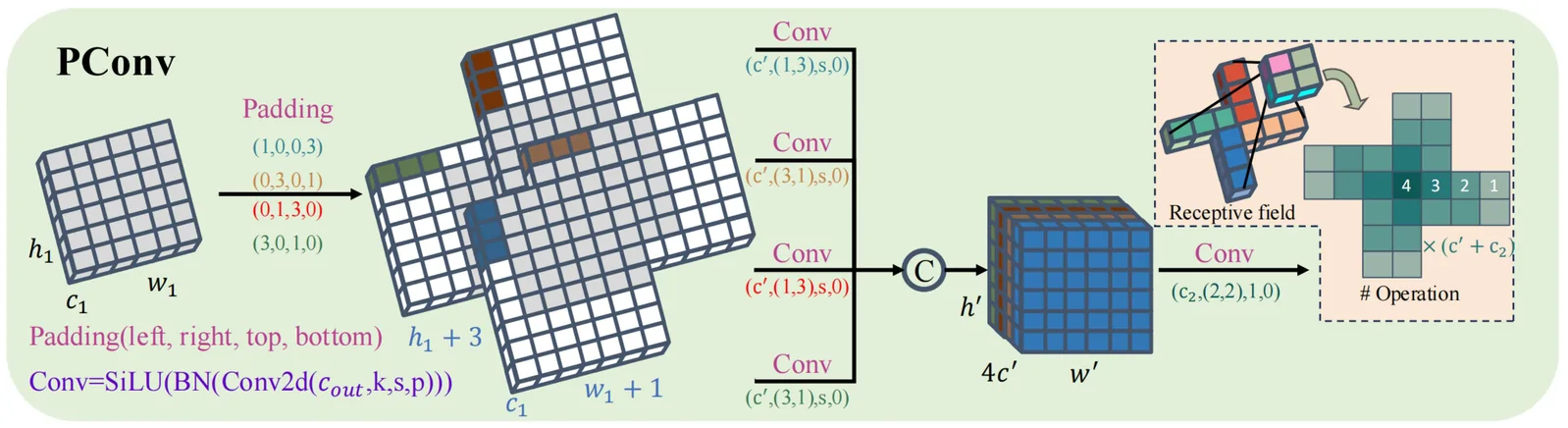
The structure of PConv.

As the core operator within the bottleneck blocks, PConv is designed to introduce direction-aware receptive fields in a lightweight manner. As illustrated in **Figure 7**, PConv processes input features through four parallel branches, each employing a specific asymmetric zero-padding scheme to bias the receptive field. The padding parameters P for each branch are formally defined in Equation 15:

(15) P∈{(1,0,0,3), (0,3,0,1), (0,1,3,0), (3,0,1,0)}

In each branch, a 3 × 3 depthwise convolution is applied to the padded input, followed by batch normalization and SiLU activation, as formulated in Equation 16:

(16) $$X_{i}=\text{SiLU}\left(\text{BN}\left(\text{Conv}2\text{d}\left({\text{Pad}}_{i}\left(X_{in}\right),\;k=3,\;s=1\right)\right)\right),\;i\in \left\{1,2,3,4\right\}$$

Notably, all branches share the same convolutional weights, effectively shifting the receptive field toward the bottom-left, top-right, top-left, and bottom-right directions with negligible parameter cost. Then, the feature maps from these four branches are concatenated along the channel dimension; a pointwise convolution and a residual connection are then applied to integrate these features, as expressed in Equation 17:

(17) $$X_{out}={\text{Conv}}_{1\times 1}\left(\text{Concat}\left[X_{1},X_{2},X_{3},X_{4}\right]\right)+X_{in}$$

This design directly overcomes the limitations of standard convolutions in capturing the anisotropic directional signals inherent to pepper anthracnose lesions. In agricultural environments, lesions exhibit distinct morphological traits across growth stages—ranging from tiny, few-pixel early-stage spots to large, irregular advanced regions with multi-directional boundary orientations. By leveraging shared-weight asymmetric sampling within PConv, C3K2_PC effectively captures multi-directional edge responses while maintaining low computational redundancy. Consequently, the neck preserves both fine-grained local details and macro structural contours during feature aggregation, seamlessly converting the enriched representations from LGMambaNet into boundary-sensitive, multi-scale feature maps without introducing substantial computational overhead.

### 2.5 DSSA module

**Figure 8:**
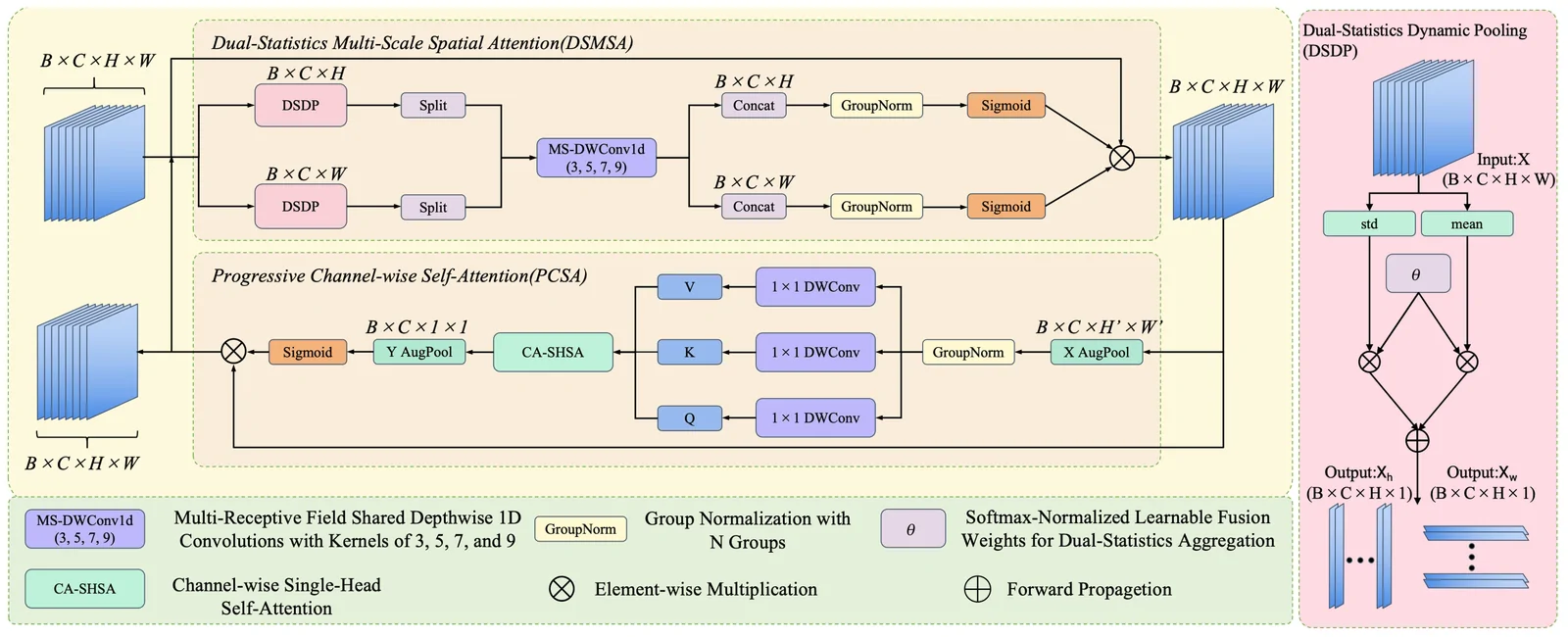
The structure of DSSA.

To further strengthen the model’s perception of lesion boundaries and texture distributions during feature fusion, this study proposes the Dual-Statistics Synergistic Attention (DSSA) module. As shown in **Figure 8**, DSSA employs a serial connection of spatial and channel attention branches to perform joint modulation on the feature maps.

#### 2.5.1 Dual-statistics multi-scale spatial attention

Unlike conventional spatial attention mechanisms that rely solely on mean pooling, DSSA introduces Dual-Statistics Dynamic Pooling (DSDP). Given input feature $X\in {\mathbb{R}}^{B\times C\times H\times W}$, the module computes both mean and standard deviation statistics along the height (*H*) and width (*W*) dimensions, respectively. Taking the *H* direction as an example, the statistics are computed as shown in Equation 18:

(18) $$\mu_{h}=\text{Mean}\left(X,\;\text{dim}=3\right),\;\sigma_{h}=\text{Std}\left(X,\;\text{dim}=3\right)$$

The two statistics are then fused via a set of learnable weights $W_{h}=\left[w_{h,0},w_{h,1}\right]$ normalized by Softmax, which is formulated as Equation 19:

(19) $$X_{h}=w_{h,0}\cdot \mu_{h}+w_{h,1}\cdot \sigma_{h},\;X_{h}\in {\mathbb{R}}^{B\times C\times H}$$

The fused feature *X_h_* is split into four channel subsets and fed into 1-D depthwise separable convolutions (DWConv-1D) with kernel sizes {3,5,7,9}, capturing multi-scale spatial dependencies ranging from local to global. Applying concatenation and the Sigmoid function, the system generates spatial weight maps $A_{h}\in {\mathbb{R}}^{B\times C\times H\times 1}$ and $A_{w}\in {\mathbb{R}}^{B\times C\times 1\times W}$ for the horizontal and vertical axes, producing the spatial branch result as expressed in Equation 20:

(20) $$X_{sa}=X⊙A_{h}⊙A_{w}$$

#### 2.5.2 Progressive channel-wise self-attention

Building upon the spatially modulated feature *X_sa_*, DSSA further models global inter-channel correlations via channel self-attention. The spatial resolution is first reduced by average pooling to lower computational cost, after which a multi-head self-attention (MHSA) mechanism operates on the channel vectors, following Equations 21, 22:

(21) $$Q,K,V={\text{Linear}}_{q,k,v}\left(\text{Pool}\left(X_{sa}\right)\right)$$

(22) $${\text{Attn}}_{ca}=\text{Softmax}\left(\frac{QK^{T}}{\sqrt{d_{k}}}\right)V$$

where *d_k_* is the key dimension. The channel attention weight $A_{ca}\in {\mathbb{R}}^{B\times C\times 1\times 1}$ is subsequently obtained via global average pooling and Sigmoid gating, and the final output is produced through a residual connection, as defined in Equation 23:

(23) $$Y=X+A_{ca}⊙X_{sa}$$

This sequential coupling strategy helps mitigate feature ambiguity between lesion regions and complex agricultural backgrounds. Specifically, DSMSA first isolates spatially salient lesion boundaries across multiple scales while suppressing background noise. Operating directly on these spatially refined representations, PCSA subsequently recalibrates inter-channel dependencies to prevent background noise from distorting global channel relationships. This progressive filtering within DSSA enhances feature discriminability for diffuse, low-contrast lesion contours while maintaining a low parameter count.

## 3 Experiment results and analysis

### 3.1 Experimental environment and parameters

Aiming for high reproducibility and scientific precision, we performed all training and assessment tasks on a unified hardware and software setup. Summarizing the specifics in **Table 2**, we provide the full configuration of the experimental platform. Using an Intel (R) Xeon (R) E5–2680 v4 processor and an NVIDIA GeForce RTX 3090 GPU (24 GB VRAM), the workstation offers enough power for the experiments. Building the model with PyTorch 2.1.0 and Python 3.10.14, the software side runs on a Linux 5.15.0 system.

**Table 2:** The configuration of the experimental platform.

Configuration	Parameter
Operating system	Linux 5.15.0-112-generic
CPU	Intel(R) Xeon(R) CPU E5–2680 v4 @ 2.40GHz
GPU	NVIDIA GeForce RTX 3090
CUDA version	11.8
cuDNN version	8.7.0
Development language	Python 3.10.14
Framework	PyTorch 2.1.0
Vision library	Torchvision 0.16.0

Hyperparameter settings were carefully optimized for different tasks and datasets to ensure the model achieved optimal convergence and performance. For the object detection tasks, including Pepper Anthracnose, Tomato Leaf, and FieldPlant, the input image size was uniformly set to 640 × 640 with a batch size of 6. The models were trained for 300 epochs on Pepper Anthracnose and FieldPlant, and 500 epochs on Tomato Leaf. For the classification task on the IP102 dataset, the input size was adjusted to 256 × 256, utilizing a larger batch size of 128 for 300 epochs. Across all tasks, the AdamW optimizer was adopted due to its superior weight decay regularization effects. The initial learning rate was configured at 2 × 10^−4^ for Pepper Anthracnose and Tomato Leaf, 1 × 10^−4^ for FieldPlant, and 1 × 10^−3^ for IP102. Detailed hyperparameter settings are provided in **Table 3**.

**Table 3:** Hyperparameter settings for different tasks and datasets.

Parameter	Pepper anthracnose	Tomato leaf	Field plant	IP102
	detection	detection	detection	classification
Image size	640×640	640×640	640×640	256×256
Epochs	300	500	300	300
Batch size	6	6	6	128
Learning rate	2×10^−4^	2×10^−4^	1×10^−4^	1×10^−3^
Optimizer	AdamW	AdamW	AdamW	AdamW
Weight decay	1×10^−4^	1×10^−4^	1×10^−4^	0.05
Betas	(0.9, 0.999)	(0.9, 0.999)	(0.9, 0.999)	(0.9, 0.999)

To evaluate the lightweight characteristics and real-time feasibility of PAD-Net for practical agricultural edge deployment, alongside the calculation of parameters and GFLOPs, the trained models were exported to the ONNX format and evaluated using TensorRT to capture performance metrics on an actual hardware platform, including frames per second (FPS), inference latency, and runtime memory footprint.

### 3.2 Comparison experiments of different models

In order to comprehensively evaluate the detection performance of PAD-Net, this study performs a thorough comparison with current state-of-the-art models under identical experimental conditions using standard COCO evaluation metrics (Lin et al., 2014). The comparison models encompass two representative paradigms: YOLO-series one-stage detectors, including YOLOv10 (Wang et al., 2024a), YOLOv12 (Tian et al., 2025), YOLOv13 (Lei et al., 2025), YOLOv26 (Sapkota et al., 2025), VajraV1 (Makkar, 2025), YOLO-Master (Lin et al., 2025), and YOLO_JD (Li et al., 2022), as well as Transformer-based end-toend detectors, including RT-DETR (Zhao et al., 2024), D-FINE (Peng et al., 2024), Deformable DETR (Zhu et al., 2020), DEIM (Huang et al., 2025b), DEIMv2 (Huang et al., 2025a), and Bio-DETR (Di et al., 2024). It is worth noting that YOLO_JD and Bio-DETR are purpose-built detectors specifically designed for agricultural vision tasks, making their inclusion in this comparison particularly meaningful for validating the practical effectiveness of PAD-Net in domain-specific scenarios. Focusing on *AP*, *AP*_50_, *AP*_75_, parameter counts, and FLOPs, this choice offers a broad review of detection quality and deployment efficiency. **Table 4** displays the specific findings of these comparative tests.

**Table 4:** Model comparison results on the pepper anthracnose dataset.

Version	Model	AP	AP_50_	AP_75_	Params (M)	GFLOPs
Nano	YOLOv10	0.392	0.803	0.330	2.71	8.4
	YOLOv12	0.419	0.853	0.370	2.57	6.5
	YOLOv13	0.398	0.806	0.311	2.50	6.5
	YOLOv26	0.381	0.780	0.326	2.57	6.1
	VajraV1	0.409	0.838	0.377	3.75	13.6
	YOLO-Master	0.421	0.840	0.373	7.55	9.9
	YOLO_JD	0.416	0.855	0.397	1.85	4.2
	RT-DETR (R18)	0.418	0.830	0.372	20.08	29.6
	DEIM	0.419	0.835	0.358	3.72	7.1
	PAD-Net (Ours)	0.430	0.846	0.382	3.99	11.5
Small	YOLOv10	0.392	0.813	0.331	8.07	24.8
	YOLOv12	0.429	0.844	0.352	9.25	21.5
	YOLOv13	0.409	0.833	0.363	9.06	21.2
	YOLOv26	0.386	0.773	0.316	9.47	20.5
	VajraV1	0.424	0.851	0.378	11.55	47.7
	YOLO-Master	0.424	0.854	0.368	29.15	34.8
	YOLO_JD	0.415	0.846	0.338	7.38	16.0
	RT-DETR (R50)	0.412	0.816	0.346	42.93	130.8
	RT-DETR (R101)	0.415	0.821	0.358	61.92	191.8
	D-FINE	0.399	0.813	0.346	10.22	24.8
	Def DETR	0.344	0.744	0.279	40.10	123.3
	DEIM	0.416	0.853	0.348	10.18	24.8
	DEIMv2	0.413	0.845	0.345	10.31	20.6
	Bio-DETR	0.417	0.840	0.387	16.16	23.0
	PAD-Net (Ours)	0.446	0.861	0.404	9.21	24.5

As shown in **Table 4**, PAD-Net achieves highly competitive performance across both the nano and small model scales, demonstrating favorable advantages in detection accuracy while maintaining competitive efficiency. At the nano scale, PAD-Net attains the highest *AP* of 0.430 among all compared models, outperforming YOLOv12 (0.419), and DEIM (0.419), while its *AP*_50_ of 0.846 remains competitive with the best-performing lightweight models. Further outperforming the Transformer-style RT-DETR (R18), our PAD-Net reaches a higher *AP* while requiring fewer parameters and FLOPs.

At the small scale, PAD-Net demonstrates even more pronounced advantages, achieving the best *AP* of 0.446 and *AP*_75_ of 0.404. These results reflect substantial gains over representative detectors. Compared to the highly competitive DEIM baseline with an *AP* of 0.416, which already outperforms most YOLO variants, PAD-Net achieves a 3.0% improvement while reducing parameters from 10.18M to 9.21M. It further surpasses YOLOv12 with an *AP* of 0.429 and DEIMv2 with an *AP* of 0.413, with the notable gain in *AP*_75_ indicating more precise lesion localization. Ultimately, PAD-Net delivers competitive accuracy with a significantly lower computational cost than RT-DETR variants (R50/R101), highlighting its practical potential for deployment on resource-constrained edge devices in agricultural settings. To assess its deployment readiness, both the Nano and Small variants of PAD-Net were evaluated using TensorRT optimization. Specifically, the Nano version achieves a throughput of 166.50 FPS, a low latency of 5.99 ms, and a compact memory footprint of 34.57 MB. Meanwhile, the Small version reaches 156.52 FPS, a latency of 6.22 ms, and a memory footprint of 46.97 MB. Notably, both variants substantially exceed the standard real-time video processing threshold of 30 FPS, effectively satisfying the demand for real-time visual feedback on edge devices in dynamic field environments. These empirical metrics demonstrate their architectural feasibility for low-latency edge processing.

Among recent small-scale YOLO variants, YOLO-Master and VajraV1 both achieve competitive *AP* values of 0.424 but remain 2.2 percentage points below PAD-Net. Moreover, YOLO-Master requires 29.15M parameters, whereas VajraV1 incurs a computational cost of 47.7GFLOPs. Conversely, YOLOv13 and YOLOv26 exhibit lower *AP* values, suggesting less suitability for fine-grained agricultural disease localization. Compared to agriculture-specific models such as YOLO_JD and Bio-DETR, PAD-Net demonstrates highly competitive performance in terms of overall average precision and operational efficiency. A holistic evaluation of these quantitative results indicates that although certain models may edge ahead in specific single-threshold indexes, PAD-Net achieves a more distinct advantage in the comprehensive *AP* metric. In practical smart farming, prioritizing the *AP* ensures a more robust bounding box regression capacity against complex field backgrounds and blurred lesion boundaries, thereby delivering a better-balanced trade-off between precision and efficiency.

**Figure 9:**
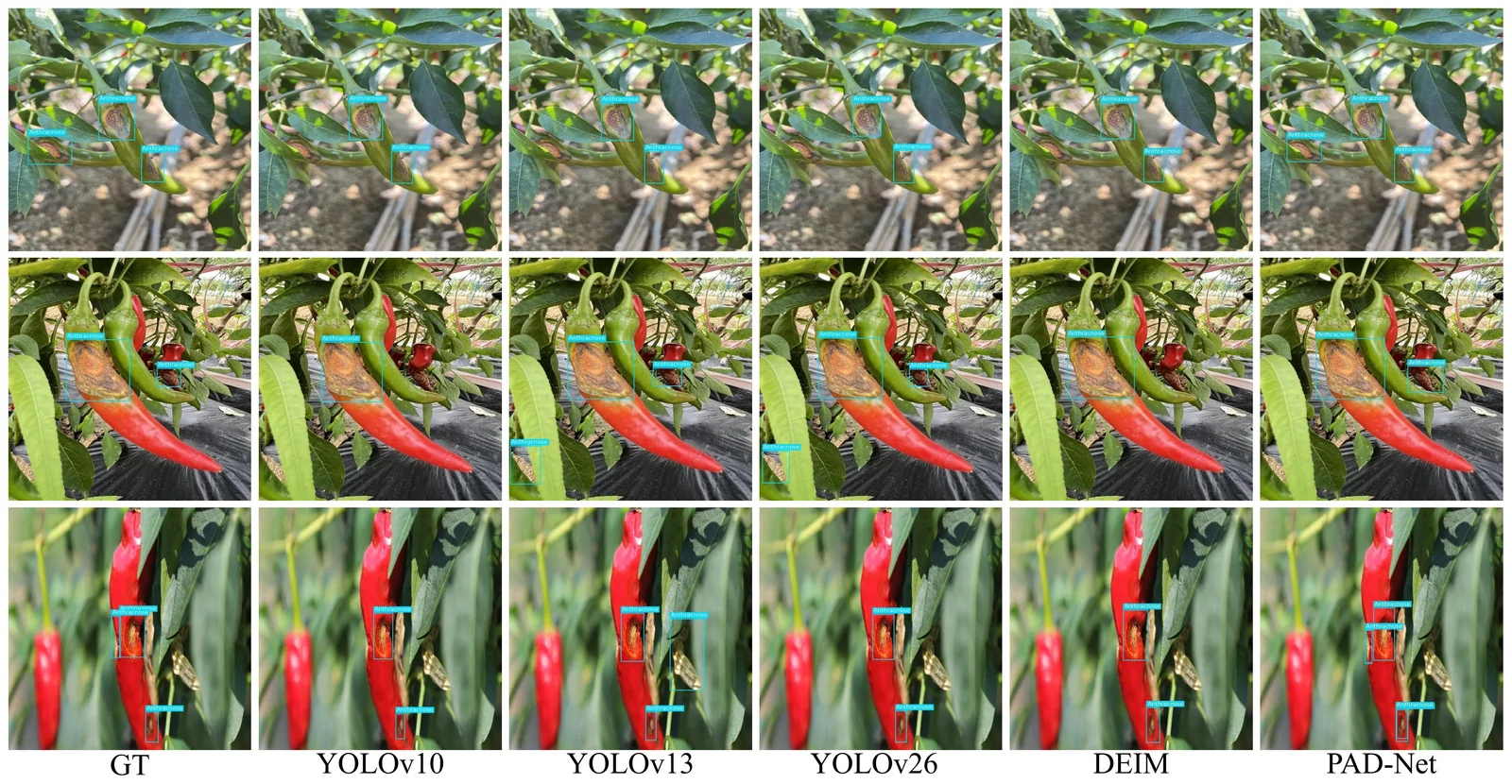
Comparison and visualization of different detection algorithms on the pepper anthracnose dataset. bounding box color indicates the target category: blue (Anthracnose).

To provide an intuitive assessment of detection quality in practical scenarios, **Figure 9** presents qualitative comparisons of bounding box predictions from representative models on challenging test samples. As observed, PAD-Net consistently produces more complete and precise detections across diverse disease manifestation scenarios, including small early-stage lesions, heavily occluded targets, and multi-instance cluttered scenes. In contrast, YOLOv10 exhibits frequent missed detections for small-scale lesions with low color contrast against the background, while YOLOv13, YOLOv26, and DEIM produce incomplete bounding boxes in occlusion-heavy scenarios. PAD-Net addresses these limitations through the LGMambaNet backbone, whose global-local hybrid modeling mechanism effectively handles partially occluded or overlapping lesions, and the DSSA attention module, which suppresses redundant background interference such as leaf texture and soil noise.

**Figure 10:**
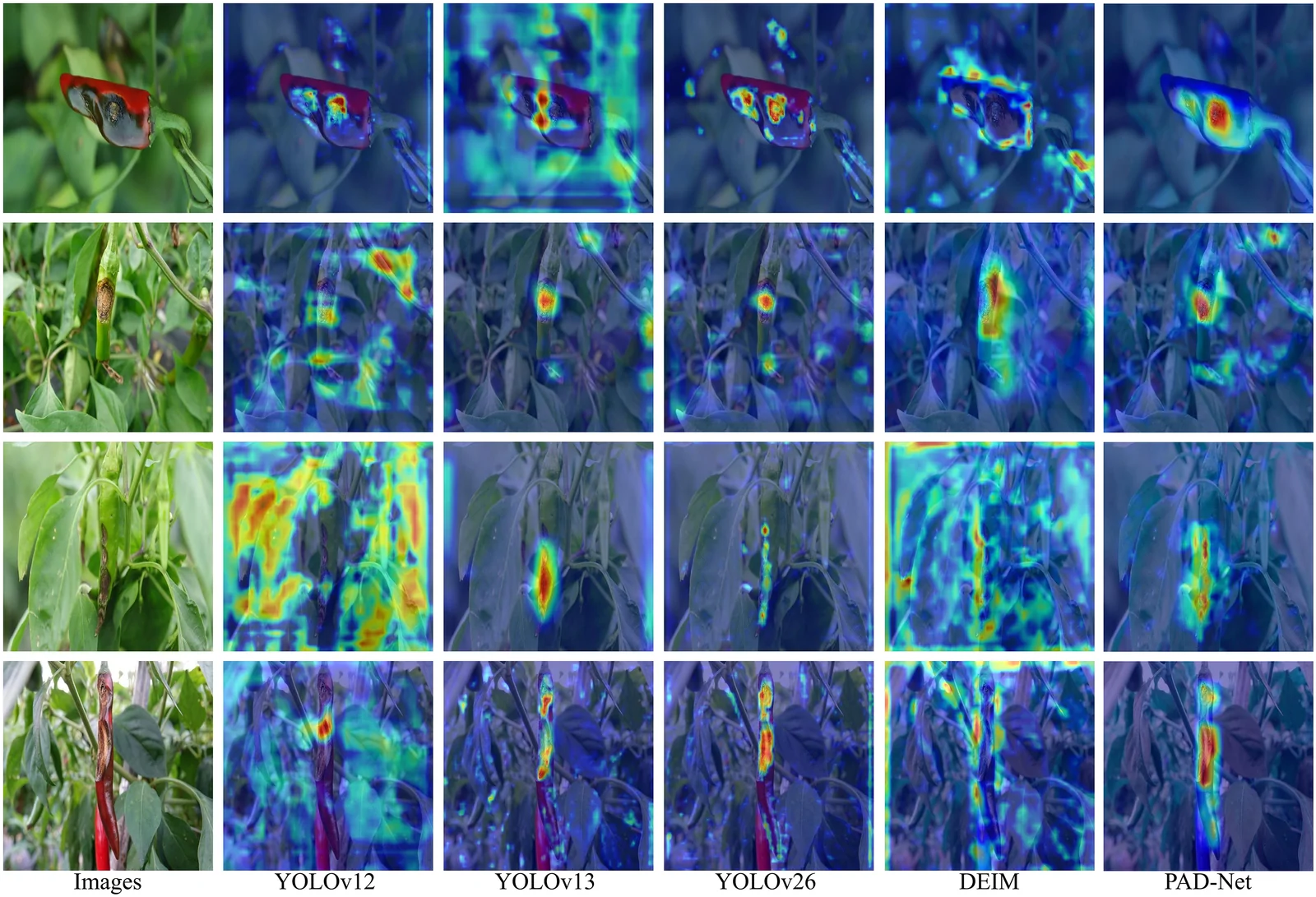
Grad-CAM heatmap visualization of different models on the pepper anthracnose dataset.

Grad-CAM visualizations in **Figure 10** further reveal that PAD-Net’s attention is accurately concentrated on diseased regions. In contrast, other models exhibit diffuse or fragmented attention maps spreading to healthy tissues or stems, reflecting limited discriminability for small-scale targets. Even when YOLOv26 and DEIM show improved localization, they struggle with small or occluded lesions under complex backgrounds. The well-focused activations of PAD-Net confirm that its feature enhancement strategy effectively guides the model toward anthracnose characteristics, reducing both false positives and missed detections in field conditions.

### 3.3 Ablation studies

To verify the effectiveness of the proposed PAD-Net and its core components in the task of pepper anthracnose detection, a series of comprehensive ablation experiments were conducted. These experiments analyze the contribution of individual modules, compare different structural designs, and optimize the backbone hyperparameters.

#### 3.3.1 Effectiveness of key modules and aggregation strategies

To verify the contribution of each proposed component to the PAD-Net framework for pepper anthracnose detection, a comprehensive ablation study was conducted. The quantitative results, including detection precision and computational complexity, are summarized in **Table 5**.

**Table 5:** Ablation study results of different module combinations on the pepper anthracnose dataset.

LGMambaNet	C3K2_PC	DSSA	AP	AP_50_	AP_75_	Params (M)	GFLOPs
–	–	–	0.416	0.853	0.348	10.18	24.8
✓	–	–	0.436	0.840	0.392	10.24	28.2
–	✓	–	0.417	0.864	0.366	9.13	21.1
–	–	✓	0.424	0.841	0.381	10.19	24.8
✓	✓	–	0.442	0.861	0.403	9.19	24.5
✓	✓	✓	0.446	0.861	0.404	9.21	24.5

The baseline model achieved an *AP* of 0.416. Upon the independent integration of the LGMambaNet backbone, the *AP* rose to 0.436, representing a significant improvement of 2.0%. This progress highlights the backbone’s proficiency in handling irregular lesion boundaries and foliage occlusion through its synergistic local fine-grained modeling and global frequency-domain analysis. In contrast, the standalone introduction of the C3K2_PC module resulted in an *AP* of 0.417 but achieved a remarkable reduction in computational cost, with parameters decreasing to 9.13 M and GFLOPs falling to 21.1. This indicates that the C3K2_PC neck, utilizing pinwheel-shaped convolutions, effectively facilitates multi-scale feature fusion and streamlines the network while maintaining high discriminative power for varied lesion scales. Similarly, the independent DSSA module elevated the *AP* to 0.424, confirming its effectiveness in suppressing complex background interference through the calibration of spatial and channel features.

The combination of LGMambaNet and C3K2_PC led to a notable performance improvement, reaching an *AP* of 0.442 with a parameter count of 9.19 M, which is lower than that of the baseline. Finally, the complete PAD-Net, incorporating all three modules, achieved the peak *AP* of 0.446. These results validate that the integrated architecture provides an optimal balance between detection precision and computational efficiency, demonstrating its strong potential for automated monitoring in smart agricultural environments.

Additionally, the aggregation strategy within the DSDP of DSSA was analyzed. As shown in **Table 6**, the “mean + std” strategy yielded the optimal *AP* of 0.446. This superiority stems from the complementary nature of the two statistics. While mean pooling captures the general regional activation level of lesion areas, standard deviation (std) measures local feature dispersion, enhancing sensitivity to subtle transitions along diffuse lesion contours. In contrast, strategies incorporating max pooling exhibit lower accuracy, as maximum values tend to react excessively to isolated noise in complex background clutter. Consequently, combining mean and std statistics strikes an effective balance between regional context representation and fine boundary discrimination.

**Table 6:** Ablation study on different aggregation strategies in the DSDP.

Method	AP	AP_50_	AP_75_
mean+max	0.429	0.858	0.359
max+std	0.436	0.859	0.384
mean+max+std	0.440	0.869	0.403
mean+std (Ours)	0.446	0.861	0.404

#### 3.3.2 Comparison with different attention, neck, and backbone mechanisms

In order to further verify the superiority of this architecture, PAD-Net is compared with various mainstream mechanisms.

As shown in **Table 7**, the DSSA attention mechanism significantly outperforms established modules such as SE (Hu et al., 2018), CA (Hou et al., 2021), CBAM (Woo et al., 2018), EMA (Ouyang et al., 2023) and SCSA (Si et al., 2025). Specifically, DSSA achieves an *AP* of 0.446, which is substantially higher than CA with an *AP* of 0.430 and EMA with an *AP* of 0.431. This superiority is primarily attributed to DSSA’s ability to effectively suppress irrelevant and complex background information—such as soil, weeds, and varying light reflections—while prominently highlighting key disease features. This demonstrates its ability to consistently focus on lesion regions rather than environmental noise in complex agricultural environments.

**Table 7:** Ablation study on different attention modules.

Method	AP	AP_50_	AP_75_	Params (M)	GFLOPs
CA	0.430	0.878	0.359	9.27	24.5
CBAM	0.443	0.872	0.417	9.39	24.5
SE	0.430	0.858	0.392	9.22	24.5
SCSA	0.438	0.872	0.379	9.21	24.5
EMA	0.431	0.849	0.380	9.23	25.8
DSSA (Ours)	0.446	0.861	0.404	9.21	24.5

In terms of the neck structure (**Table 8**), C3K2_PC demonstrates a better balance between detection accuracy and model complexity compared to RepNCSPELAN4 and DSC3K2. While DSC3K2 offers a lower parameter count, its *AP* of 0.427 falls notably short of the 0.446 achieved by C3K2_PC.

**Table 8:** Ablation study on different neck components.

Method	AP	AP_50_	AP_75_	Params (M)	GFLOPs
RepNCSPELAN4	0.430	0.861	0.356	10.25	28.2
C3K	0.428	0.847	0.385	9.75	25.9
C3K2	0.428	0.843	0.375	9.13	23.0
DSC3K2	0.427	0.849	0.406	8.85	21.5
C3K2_PC (Ours)	0.446	0.861	0.404	9.21	24.5

Finally, the LGMambaNet backbone was compared against several state-of-the-art backbones. As summarized in **Table 9**, LGMambaNet achieves the highest *AP*_50_ of 0.861 and *AP* of 0.446. Compared to the lightweight MobileViT, LGMambaNet not only improves *AP* by 2.8% but also maintains a significantly lower computational cost of 24.5GFLOPs against 43.2GFLOPs.

**Table 9:** Comparison with different backbone networks.

Method	AP	AP_50_	AP_75_	Params (M)	GFLOPs
UniConvNet-A	0.422	0.851	0.404	11.72	26.0
MobileViT-XS	0.418	0.859	0.362	8.97	43.2
EfficientViM-M1	0.365	0.745	0.321	12.9	32.8
LGMambaNet (Ours)	0.446	0.861	0.404	9.21	24.5

#### 3.3.3 Backbone structural optimization

To determine the optimal configuration for LGMambaNet, ablation experiments were performed on three key architectural dimensions: global/local ratios (*ξ,µ*), channel dimensions (*C*), and block depths (*D*).

According to **Table 10**, the ratio configuration of *ξ* = {0.8,0.8,0.7,0.6} and *µ* = {0.2,0.2,0.2,0.3} provided the best detection performance, indicating that a higher emphasis on global context in early stages is beneficial for pepper disease detection. In terms of scaling, the dimension *C* = {48,64,96,128} and depth *D* = {2,3,7,2} were selected as the final configuration. While deeper or wider networks (e.g., *D* = {2,4,9,3}) slightly improved certain metrics, they resulted in a substantial increase in parameters and GFLOPs without a proportional gain in *AP*. Thus, this experiment ultimately selected the configuration of *ξ* = {0.8,0.8,0.7,0.6}, *µ* = {0.2,0.2,0.2,0.3}, *C* = {48,64,96,128}, and *D* = {2,3,7,2}.

**Table 10:** Ablation study on backbone hyperparameters.

Configuration	AP	AP_50_	AP_75_	Params (M)	GFLOPs
Ablations on global *ξ* and local *μ* ratios					
ξ={0.6,0.6,0.6,0.5},μ={0.4,0.3,0.3,0.3}	0.435	0.843	0.371	9.16	24.6
ξ={0.7,0.7,0.6,0.5},μ={0.2,0.2,0.3,0.3}	0.433	0.848	0.400	9.16	24.6
ξ={0.8,0.8,0.7,0.6},μ={0.2,0.2,0.2,0.3} (Ours)	0.446	0.861	0.404	9.21	24.5
Ablations on dimensions *C*					
C={40,56,80,112}	0.439	0.872	0.401	8.73	22.3
C={48,64,96,128} (Ours)	0.446	0.861	0.404	9.21	24.5
C={56,80,112,144}	0.440	0.856	0.382	10.09	29.2
Ablations on depths *D*					
D={1,2,6,2}	0.434	0.863	0.417	8.91	21.7
D={2,3,7,2} (Ours)	0.446	0.861	0.404	9.21	24.5
D={2,4,9,3}	0.443	0.867	0.428	9.91	26.2

Overall, across all ablation experiments, although variants may occasionally yield better results on single-threshold metrics, the final configuration of PAD-Net is selected due to its consistent performance and robustness across experimental dimensions. Achieving the highest *AP*, this configuration delivers a balanced detection capability when handling complex field backgrounds and blurred lesion boundaries.

### 3.4 Generalization performance on external agricultural datasets

To further assess PAD-Net’s robustness and cross-crop transferability, generalization experiments were conducted on the Tomato Leaf and FieldPlant datasets (Imtiaz et al., 2025; Moupojou et al., 2023).

As summarized in **Tables 11**, **12**, PAD-Net demonstrates consistently strong generalization at both nano and small scales. On Tomato Leaf, PAD-Net-N achieves the highest *AP* of 0.484, while PAD-Net-S attains an *AP* of 0.490 and an *AP*_75_ of 0.535. The latter also exceeds the *AP* of the much larger RT-DETR (R101), despite using only 9.21M parameters compared with 76.51M. On FieldPlant, PAD-Net-N reaches an *AP* of 0.478 and an *AP*_75_ of 0.522, improving the *AP* of DEIM-N by 2.0 percentage points. PADNet-S further obtains the highest overall *AP* of 0.487 and an *AP*_75_ of 0.529 with 9.23M parameters. The consistent accuracy gains on both external datasets demonstrate that PAD-Net preserves a favorable balance between detection precision and model complexity across different agricultural detection tasks.

**Table 11:** Model comparison results on the tomato leaf dataset.

Version	Model	AP	AP_50_	AP_75_	Params (M)	GFLOPs
Nano	YOLOv9	0.464	0.715	0.483	2.13	8.5
	YOLOv10	0.428	0.613	0.472	2.78	8.7
	YOLOv11	0.458	0.724	0.454	2.62	6.6
	YOLOv13	0.452	0.708	0.410	2.49	6.5
	YOLOv26	0.360	0.565	0.368	2.57	6.1
	YOLO-Master	0.430	0.666	0.464	7.55	9.9
	YOLO_JD	0.416	0.719	0.391	1.87	4.3
	RT-DETR (R18)	0.448	0.680	0.477	20.09	29.6
	DEIM	0.473	0.742	0.502	3.73	7.1
	PAD-Net (Ours)	0.484	0.733	0.517	3.99	11.5
Small	YOLOv9	0.477	0.712	0.499	7.32	27.6
	YOLOv10	0.456	0.673	0.509	8.13	25.1
	YOLOv11	0.465	0.742	0.463	9.46	21.7
	YOLOv13	0.461	0.711	0.453	9.06	21.2
	YOLOv26	0.433	0.652	0.468	10.01	22.8
	YOLO-Master	0.462	0.722	0.507	29.12	34.6
	YOLO_JD	0.462	0.736	0.508	7.41	16.3
	RT-DETR (R50)	0.465	0.718	0.455	42.79	67.9
	RT-DETR (R101)	0.487	0.739	0.506	76.51	129.4
	D-FINE	0.469	0.736	0.481	10.23	24.6
	Def DETR	0.463	0.745	0.499	40.10	123.3
	Bio-DETR	0.439	0.732	0.484	16.16	23.0
	DEIM	0.461	0.687	0.490	10.18	24.8
	DEIMv2	0.463	0.741	0.401	10.28	20.3
	PAD-Net (Ours)	0.490	0.736	0.535	9.21	24.5

**Table 12:** Model comparison results on the FieldPlant dataset.

Version	Model	AP	AP_50_	AP_75_	Params (M)	GFLOPs
Nano	YOLOv11	0.459	0.579	0.495	2.62	6.6
	YOLOv12	0.421	0.565	0.467	2.60	6.7
	YOLOv13	0.427	0.554	0.470	2.49	6.5
	YOLOv26	0.439	0.577	0.469	2.57	6.1
	VajraV1	0.316	0.437	0.345	3.76	13.6
	YOLO-Master	0.402	0.518	0.439	7.55	9.9
	YOLO_JD	0.302	0.492	0.316	1.90	4.4
	D-FINE	0.424	0.531	0.454	3.75	3.7
	DEIM	0.458	0.572	0.489	3.73	7.1
	PAD-Net (Ours)	0.478	0.584	0.522	3.99	11.6
Small	YOLOv11	0.474	0.605	0.521	9.46	21.7
	YOLOv12	0.448	0.583	0.485	9.28	21.7
	YOLOv13	0.453	0.570	0.489	9.06	21.2
	YOLOv26	0.476	0.637	0.504	10.01	22.8
	VajraV1	0.413	0.543	0.443	11.56	47.8
	YOLO-Master	0.450	0.569	0.492	29.15	34.8
	YOLO_JD	0.350	0.541	0.385	7.48	16.5
	D-FINE	0.436	0.551	0.477	10.23	24.6
	Bio-DETR	0.478	0.622	0.515	16.16	23.0
	DEIM	0.460	0.567	0.500	10.20	25.0
	DEIMv2	0.395	0.486	0.426	10.28	20.4
	PAD-Net (Ours)	0.487	0.613	0.529	9.23	24.6

**Figure 11:**
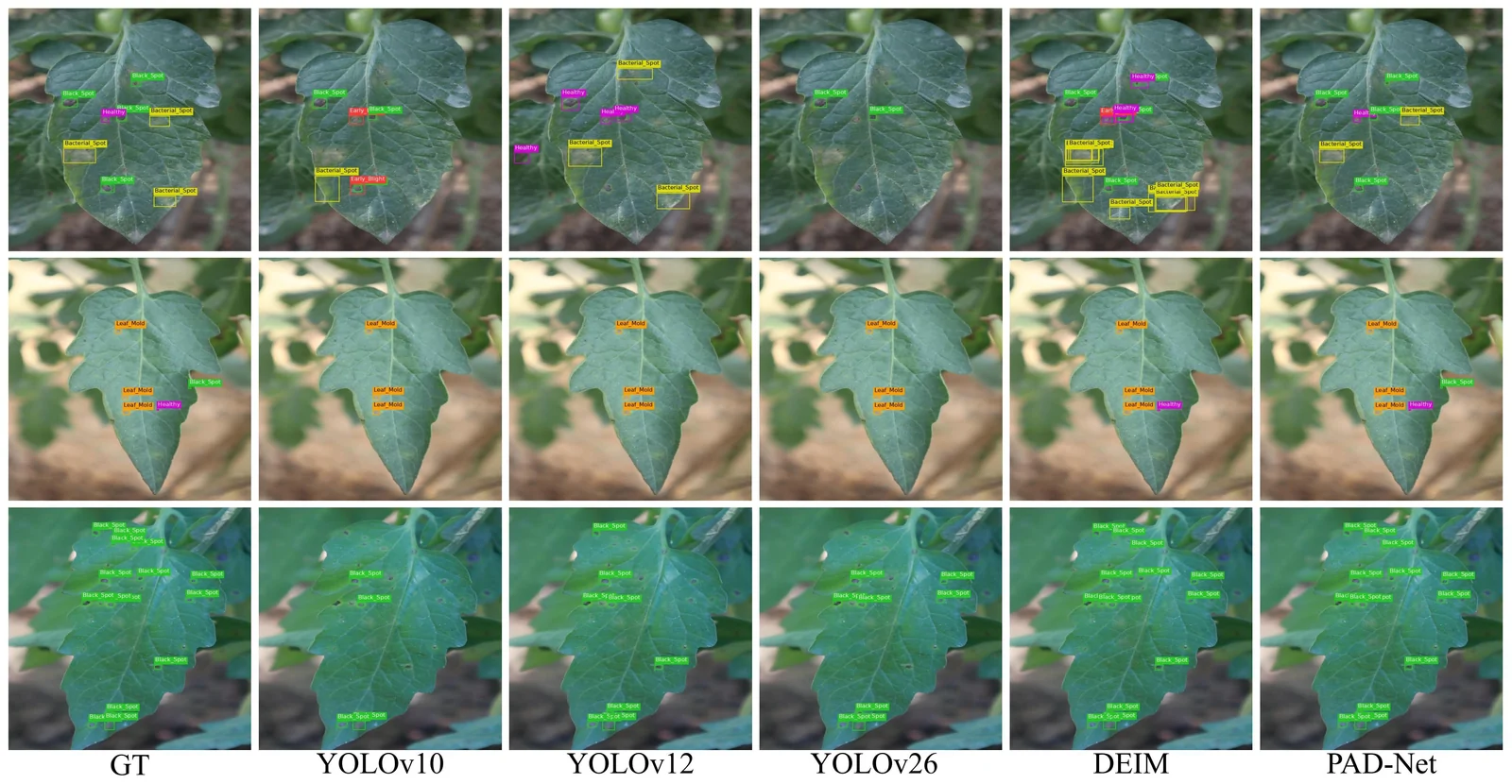
Comparison and visualization of different detection algorithms on Tomato Leaf Dataset. Bounding box colors indicate different categories: red (*Early Blight*), green (*Black Spot*), orange (*Leaf Mold*), yellow (*Bacterial Spot*), and magenta (*Healthy*).

**Figure 12:**
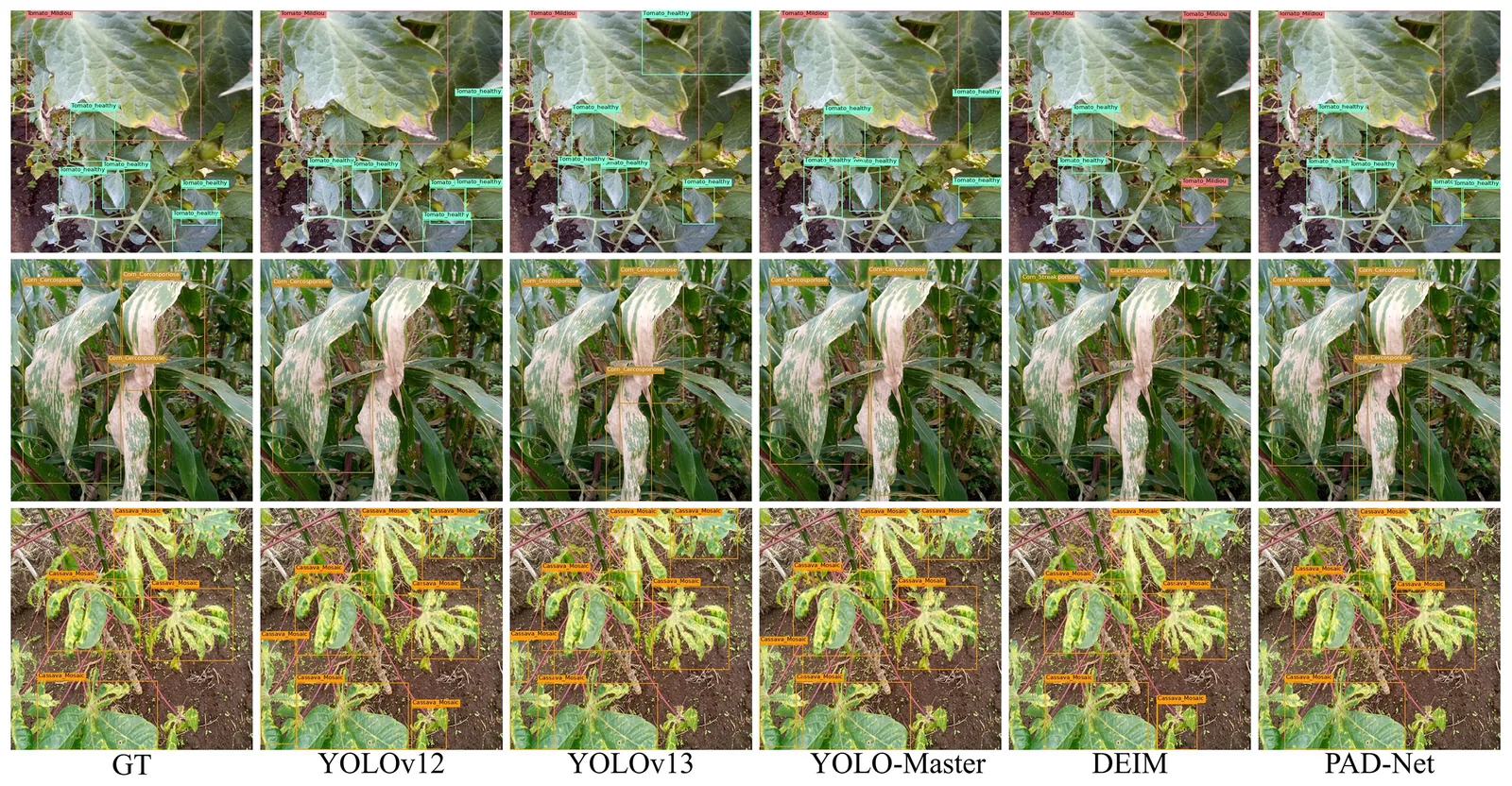
Comparison and visualization of different detection algorithms on the FieldPlant dataset. Bounding box colors indicate different categories: aquamarine (*Tomato Healthy*), red (*Tomato Mildiou*), brownish yellow (*Corn Cercosporiose*), and orange (*Cassava Mosaic*).

**Figures 11**, **12** present qualitative comparisons on the Tomato Leaf and FieldPlant datasets, respectively. On Tomato Leaf, while other detectors suffer from missed detections of small or lowcontrast lesions, false positives, or confusion between visually similar categories, PAD-Net produces more complete detections that closely align with the ground-truth annotations. In FieldPlant’s dense, highoverlap scenes with partial occlusion and complex natural backgrounds, PAD-Net maintains more complete target localization and consistent category predictions. Together with the ablation results, these visual comparisons support the effectiveness of global–local feature modeling and background suppression in preserving key disease cues while filtering out complex textures and background noise, thereby improving detection reliability for challenging samples.

Overall, the quantitative results and qualitative observations on these two external datasets provide further evidence that PAD-Net achieves consistently competitive detection performance in complex agricultural scenes. Its performance across different crops, disease categories, target appearances, and background conditions also indicates favorable applicability and practical deployment potential across diverse agricultural detection tasks.

### 3.5 Generalization assessment on agricultural classification

To further demonstrate the robust feature extraction capability and cross-domain transferability of the designed LGMambaNet backbone, we conducted extension experiments on the IP102 dataset (Wu et al., 2019). As a large-scale benchmark dataset for pest identification, IP102 has a complex environmental background, providing a rigorous environment for verifying the efficient ability of the backbone network serving as the foundation of PAD-Net in various agricultural visual tasks.

As shown in **Table 13**, LGMambaNet achieved a Top-1 accuracy of 69.58%, significantly outperforming various CNN, Transformer, and Mamba-based architectures (Mehta and Rastegari, 2021; Lee et al., 2025; Howard et al., 2019; Maaz et al., 2022; Shaker et al., 2023; Tan and Le, 2021; Xie et al., 2024). Specifically, compared to the classic CNN-based MobileNetV3-Large, our backbone achieves superior Top-1 accuracy using only 2.34M parameters, whereas the latter requires 4.33M parameters. Furthermore, LGMambaNet demonstrated a clear advantage over state-of-the-art Mamba variants, surpassing EfficientViM-M2 by 6.28% and QuadMamba-Li by 5.78%. These results highlight that the hybrid global-local modeling mechanism of LGMambaNet effectively captures essential morphological cues even under the high intra-class variance typical of real-world agricultural scenes.

**Table 13:** Backbone comparison results on the IP102 classification dataset.

Model	Top-1 (%)	Top-5 (%)	Precision	Recall	F1-score	Params (M)	GFLOPs
EdgeNeXt-XSmall	62.95	83.08	60.92	55.88	57.89	2.16	0.413
MobileNetV3-Large	66.49	86.77	62.66	59.29	60.64	4.33	0.224
EfficientNetV2-B0	58.20	83.36	53.56	47.58	49.34	5.99	0.728
MobileViT-S	63.72	86.10	58.60	54.09	55.63	5.00	1.441
SwiftFormer-XS	64.44	84.77	60.88	57.18	58.69	3.06	0.605
EfficientViM-M2	63.30	81.55	62.24	55.79	58.37	12.54	0.354
QuadMamba-Li	63.80	81.69	61.17	56.36	58.28	5.36	0.847
LGMambaNet (Ours)	69.58	89.47	65.16	60.47	62.12	2.34	0.602

**Figure 13:**
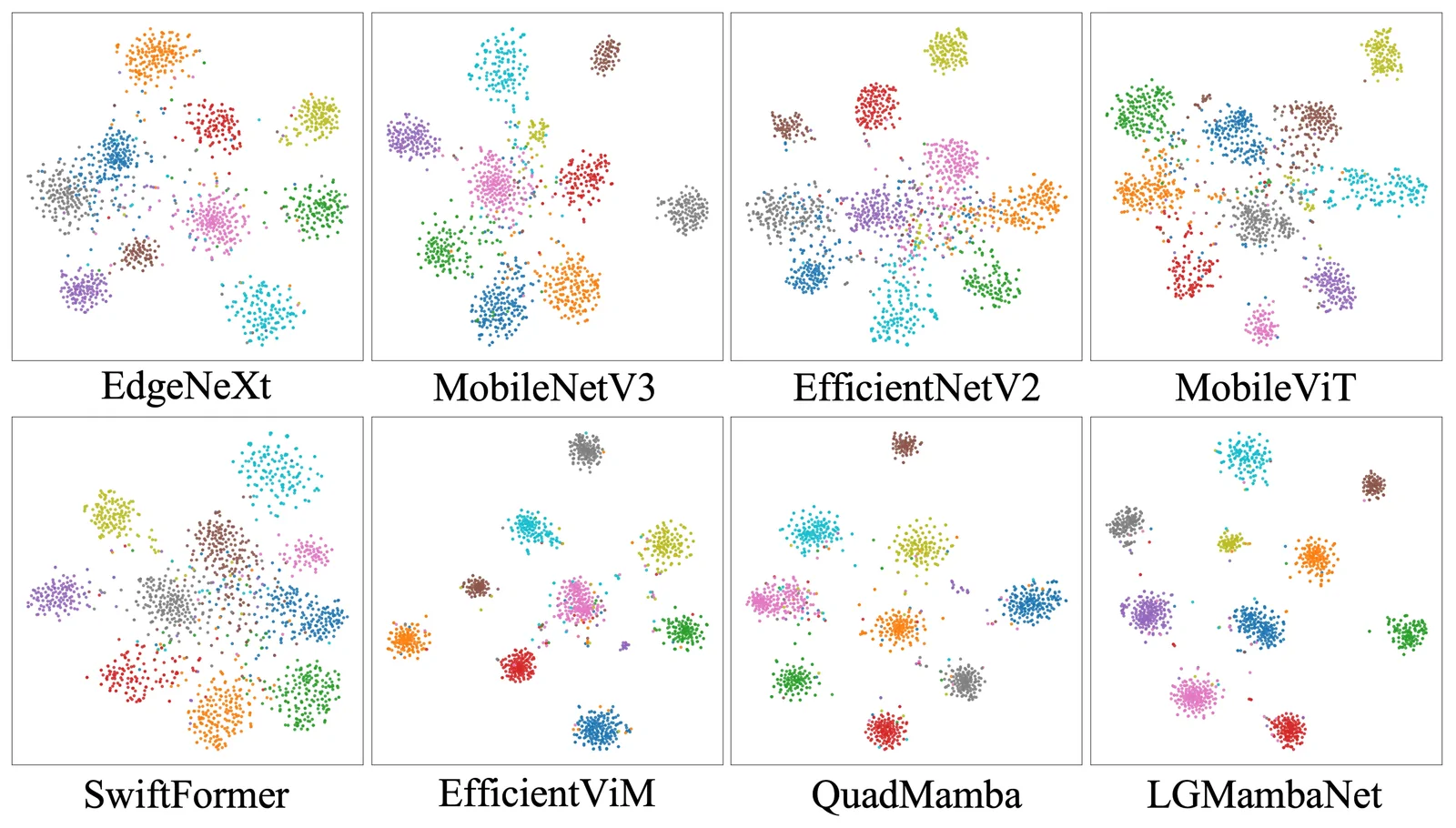
t-SNE visualization of the feature embedding space on the IP102 dataset for different models.

**Figure 14:**
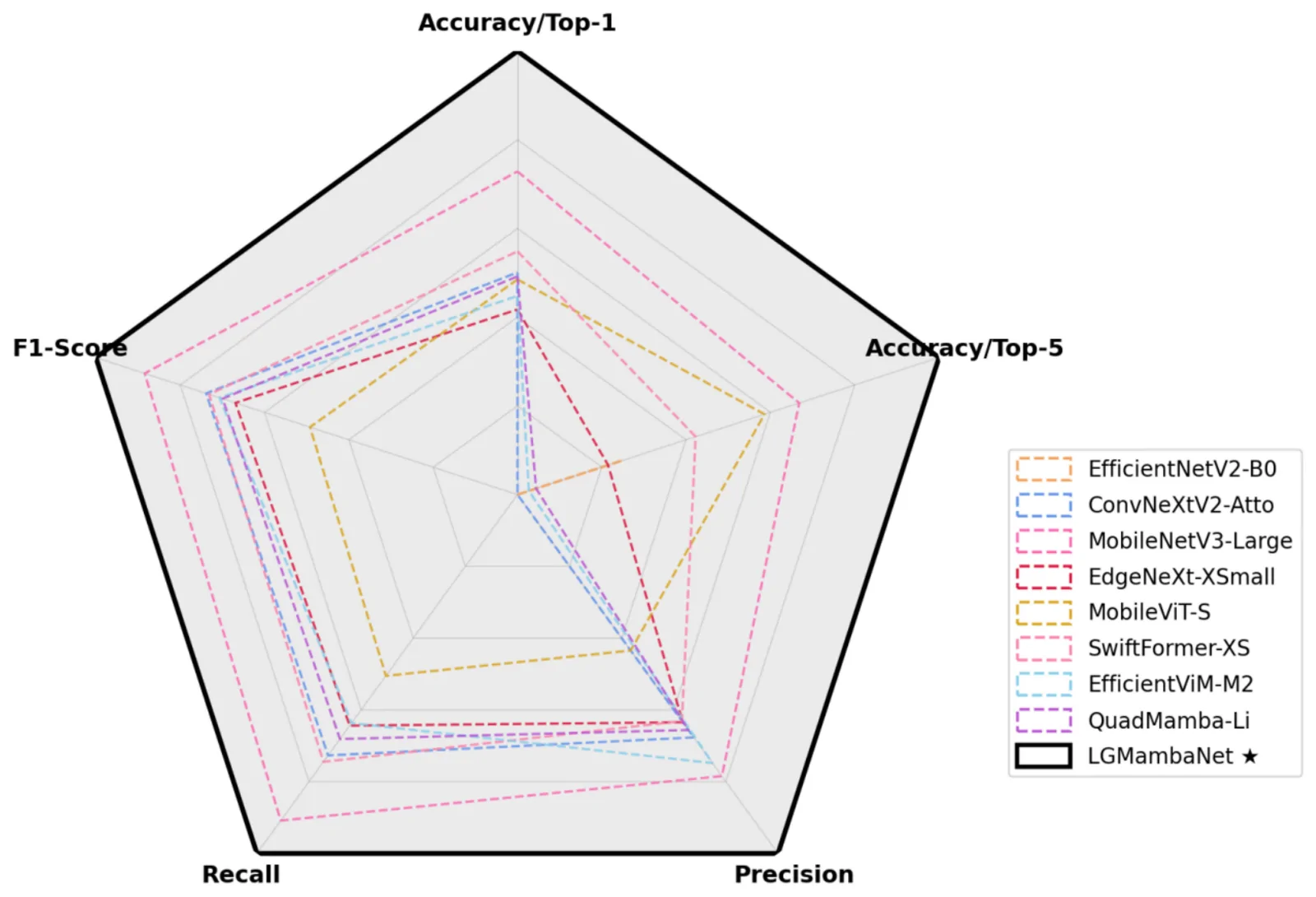
Radar chart visualizing the multi-dimensional performance of different models on the IP102.

To further examine the feature representation learned by LGMambaNet, **Figure 13** presents a t-SNE visualization of the feature embedding space on the IP102 dataset. Compared with other mainstream architectures, LGMambaNet produces more compact intra-class clusters and clearer inter-class separation, indicating that its hybrid global–local modeling mechanism learns discriminative representations for agricultural pest classification. In addition, the radar chart in **Figure 14** summarizes the multidimensional performance of the compared models and demonstrates that LGMambaNet achieves a favorable balance among classification accuracy, parameter efficiency, and computational complexity. This consistent performance across both detection and classification tasks underscores the potential of PAD-Net for deployment on resource-constrained agricultural monitoring devices.

## 4 Discussion

The proposed PAD-Net algorithm achieves competitive performance in detecting pepper anthracnose under complex agricultural environments, demonstrating clear advantages over existing object detection methodologies. Facing challenging issues such as irregular lesion boundaries, drastic cross-scale variations, and leaf occlusion, conventional algorithms perform poorly, whereas the end-to-end PAD-Net introduced in this study significantly improves detection precision and boundary localization capability. In terms of parameter size and computational overhead, the classic RT-DETR-R101 model requires 61.92M parameters and 191.8GFLOPs with an AP of 0.415, whereas the small version of PAD-Net achieves a superior AP of 0.446, representing a 3.0% improvement over the DEIM baseline, with only 9.21M parameters and 24.5GFLOPs. Meanwhile, under TensorRT benchmarks on the evaluated hardware platform, the model attains a throughput of 156.52FPS and a low latency of 6.22ms, demonstrating its real-time inference capability and indicating potential for future deployment on mobile and embedded devices.

Regarding technical innovations, the self-designed LGMambaNet backbone synergizes local fine-grained modeling via the deformable sliding window of DSwin-Mamba with global frequency-domain analysis using the Haar wavelet transforms of FA-WTMamba, addressing feature loss in complex canopies. The C3K2_PC neck module leverages Pinwheel-shaped Convolutions, also known as PConv, with four-way asymmetric sampling, capturing multi-directional edge signals while reducing parameter overhead by 0.97M compared to the baseline. Furthermore, the DSSA module combines Dual-Statistics Dynamic Pooling utilizing mean and standard deviation with progressive channel self-attention to effectively suppress interference from foliage textures, soil backgrounds, and varying light reflections.

Additionally, cross-crop generalization experiments on Tomato Leaf and FieldPlant datasets, alongside classification benchmarks on the IP102 dataset, were conducted as supporting evidence to evaluate the representation capability and applicability of the architecture across different agricultural vision tasks. PAD-Net exhibited impressive cross-crop detection performance and strong generalization ability on both Tomato Leaf and FieldPlant datasets. Moreover, the LGMambaNet backbone attained a Top-1 accuracy of 69.58% with only 2.34M parameters on IP102, outperforming state-of-the-art Mamba baselines such as EfficientViM-M2 at 63.30%, which confirms the universal feature representation capability of our framework.

Despite these promising results, several limitations remain in the present study. First, the self-built dataset is relatively limited in disease categories, focusing exclusively on pepper anthracnose. Second, deployment and execution verification on low-power embedded devices have not yet been realized. Thirdly, the antiinterference ability in complex natural environment is lack of specific experiments, and the reliability of operation under harsh conditions needs to be further verified. To address these deficiencies, future work will expand pepper datasets to cover multiple cultivars and pathogens, advance model deployment and performance optimization on low-power edge devices, and focus on integrating multi-modal data such as multispectral imaging and environmental sensors to significantly enhance detection stability and anti-interference capability in complex natural environments.

## 5 Conclusions

This study proposed PAD-Net, an end-to-end detector designed for pepper anthracnose detection in complex agricultural environments. To tackle key challenges such as irregular lesion boundaries, target occlusion, severe scale variations, and background noise, the framework incorporates three core design components. The LGMambaNet backbone synergizes local fine-grained modeling with global frequency-domain analysis to construct robust feature representations. The C3K2_PC neck facilitates multi-scale feature fusion while capturing multi-directional edge signals through pinwheel-shaped convolutions. Furthermore, the DSSA module recalibrates spatial and channel features to effectively suppress complex background interference.

Experimental results demonstrate that PAD-Net achieves a favorable balance between detection accuracy and computational efficiency. Evaluations on the external Tomato Leaf and FieldPlant datasets further show that PAD-Net maintains competitive detection performance across different crops, disease categories, target appearances, and complex background conditions. In addition, the classification results on IP102 provide further evidence of the effective feature representation capability of the LGMambaNet backbone across agricultural vision tasks. Overall, this study presents a promising algorithmic framework for automated crop disease detection, offering both theoretical value and practical significance.

## Data Availability

The raw data supporting the conclusions of this article will be made available by the authors, without undue reservation.
